# Integrated GIS-based multi-criteria and geospatial approach for seismic hazard mapping in the arabian–eurasian convergence zone

**DOI:** 10.1038/s41598-026-67303-5

**Published:** 2026-08-19

**Authors:** Kaifi Chomani, Shaki Pshdari, Rawshan Ali, Dinesh Kumar Vishwakarma, Amnah Aldohan, Ozgur Kisi, Youssef M. Youssef, Faten Nahas

**Affiliations:** 1 Department of Civil Engineering, College of Engineering, University of Raparin, Ranya, 46012 Iraq; 2 Department of Civil Engineering, Graphic Era Deemed to be University, 566/6, Bell Road, Society Area, Clement Town, Dehradun, Uttarakhand, 248002 India; 3 Department of Geography and Environmental Sustainability, College of Humanities and Social Sciences, Princess Nourah bint Abdulrahman University, P.O. Box 84428, Riyadh, 11671 Saudi Arabia; 4 Department of Civil Engineering, Technische Hochschule Lübeck, 23562 Lübeck, Germany; 5 Department of Civil Engineering, Ilia State University, Tbilisi, 0162 Georgia; 6 School of Civil, Environmental and Architectural Engineering, Korea University, Seoul, 02841 South Korea; 7 Geological and Geophysical Engineering Department, Faculty of Petroleum and Mining Engineering, Suez University, Suez, 43518 Egypt; 8 Department of Geography, College of Humanities and Social Sciences, King Saud University, Riyadh, 11451 Saudi Arabia

**Keywords:** Seismic Hazard Zonation, Geographic Information Systems, Analytic Hierarchy Process, Frequency Ratio; Earthquake Risk Assessment, Environmental sciences, Natural hazards, Solid Earth sciences

## Abstract

Earthquakes represent one of the most destructive natural hazards, particularly in developing regions along the convergence zone of the Arabian and Eurasian tectonic plates, where they result in severe human casualties, extensive infrastructure damage, and major economic losses. This study applies an integrated methodology that combines geospatial data with a Geographic Information Systems (GIS)-based Analytic Hierarchy Process (AHP) to produce the first regional-scale, screening-level Seismic Hazard Zonation (SHZ) map for the Kurdistan Region of Iraq (KRI), situated at this tectonic boundary. Eight governing factors, including distance to active faults, tectonic lineaments, lithological characteristics, soil properties, earthquake magnitude, seismic frequency, and focal depth, were analyzed and weighted according to their relative influence on seismic hazard potential. Validation using the Frequency Ratio (FR) approach demonstrated FR values below 1 in low-susceptibility areas, approximately 1 in moderate-hazard zones, above 1 in the high hazard class (FR = 1.66), and markedly elevated in the very high hazard class (FR = 9.27), indicating a positive spatial association between the modeled hazard distribution and recorded seismic events; the latter value is partly inflated by the small areal extent of the very high class and the spatial clustering of events within it. The analysis categorized KRI into five hazard levels: very low, low, moderate, high, and very high. Approximately 2,284 km^2^ (4.9%) of the region falls within the very high hazard class, highlighting the urgent need for site-specific mitigation measures and disaster preparedness strategies. Overall, this screening-level relative zonation provides a first-order spatial prioritization of seismic hazard in the KRI, supporting regional planning and guiding policymakers toward areas where detailed site-specific investigations, probabilistic seismic hazard analysis, and geotechnical characterization should be prioritized.

## Introduction

Earthquakes are among the most destructive natural disasters, significantly altering the Earth’s surface in affected regions and causing extensive loss of life, infrastructure damage, and severe economic repercussions^1–3^. Seismic activities profoundly impact topography, sediment dynamics, and erosion processes^4,5^. These hazards arise from the sudden release of energy within the Earth’s crust, generating seismic waves and ground shaking. Friction along fault lines, driven by tectonic plate movements, serves as the primary trigger for seismic activity^6,7^. Historical data indicate that approximately 8.5 million fatalities and $4.3 billion in economic losses were associated with over 13,000 catastrophic earthquake events^4,8,9^.

Seismic Hazard Zonation (SHZ) mapping constitutes a fundamental component of hazard assessment, providing critical information for urban development planning and the formulation of disaster mitigation strategies^10,11^. Recent advancements have facilitated the development of probabilistic seismic hazard maps for tectonic collision zones globally, including Italy^12^, Turkey^13^, United States of America^14^, European Union^15^. Analyzing seismic hazards is essential for disaster mitigation, risk management, and emergency preparedness^16^. Incorporating seismic hazard considerations into the design of structures and facilities is vital for minimizing risks and enhancing resilience^17^.

A robust SHZ assessment requires the integration of diverse geological, geophysical, and seismological parameters^18^. Consequently, policymakers, engineers, and urban planners must incorporate natural hazard evaluations into land-use planning processes to support sustainable territorial management and safeguard human populations. Consequently, policymakers, engineers, and urban planners must incorporate natural hazard evaluations into land-use planning processes to support sustainable territorial management and safeguard human populations^19,20^. In this context, SHA provides urban planners with critical information that supports the reduction of seismic risk and the mitigation of potential earthquake^21^.

Advances in SHZ have been achieved through integrating multiple approaches. Geographic Information System (GIS) technologies have proven invaluable for managing and analyzing complex datasets^4^. The combination of GIS and geospatial techniques is widely used for disaster-related hazard zonation^22^. Multi-Criteria Decision Analysis (MCDA) is another effective method, often integrated with GIS, to process complex decisions and assess multiple hazard parameters. Among MCDA tools, the Analytic Hierarchy Process (AHP) has been extensively applied to improve hazard assessments^23^. The considered parameters should be ranked and weighted reasonably according to their impact and importance on the hazard^24^. Additionally, the Fuzzy Analytic Hierarchy Process (FAHP) has been employed for SHZ in regions such as Iran^25^.

In Middle Asia region, one of the most significant earthquakes in Iraq occurred in November 2017 along the Iraq-Iran border, with a magnitude of 7.3. This event caused 530 fatalities in Iran, nine in the Kurdistan Region of Iraq (KRI), and over 550 injuries^26–28^. Significant damage was recorded in Halabja and nearby areas, highlighting the region’s seismic vulnerability^28^. Various studies have focused on seismic hazard assessments within the KRI, such as the tectonic analysis of the Shaikhan earthquakes^29^, seismic analysis in Duhok city^30,31^, seismic activity hazard assessment in Koya city^4^, AbdulJaleel & Taha^32^ indicated that many buildings in Erbil city are expected to experience severe damage during strong earthquakes due to non-compliance with seismic standards^33,34^. Remarkably, the KRI lies along the convergence zone of the Arabian and Eurasian tectonic plates^35,36^, where northward movement of the Arabian Plate results in subduction beneath the Eurasian Plate, making the region one of the most seismically active globally^37^.

**Fig. 1:**
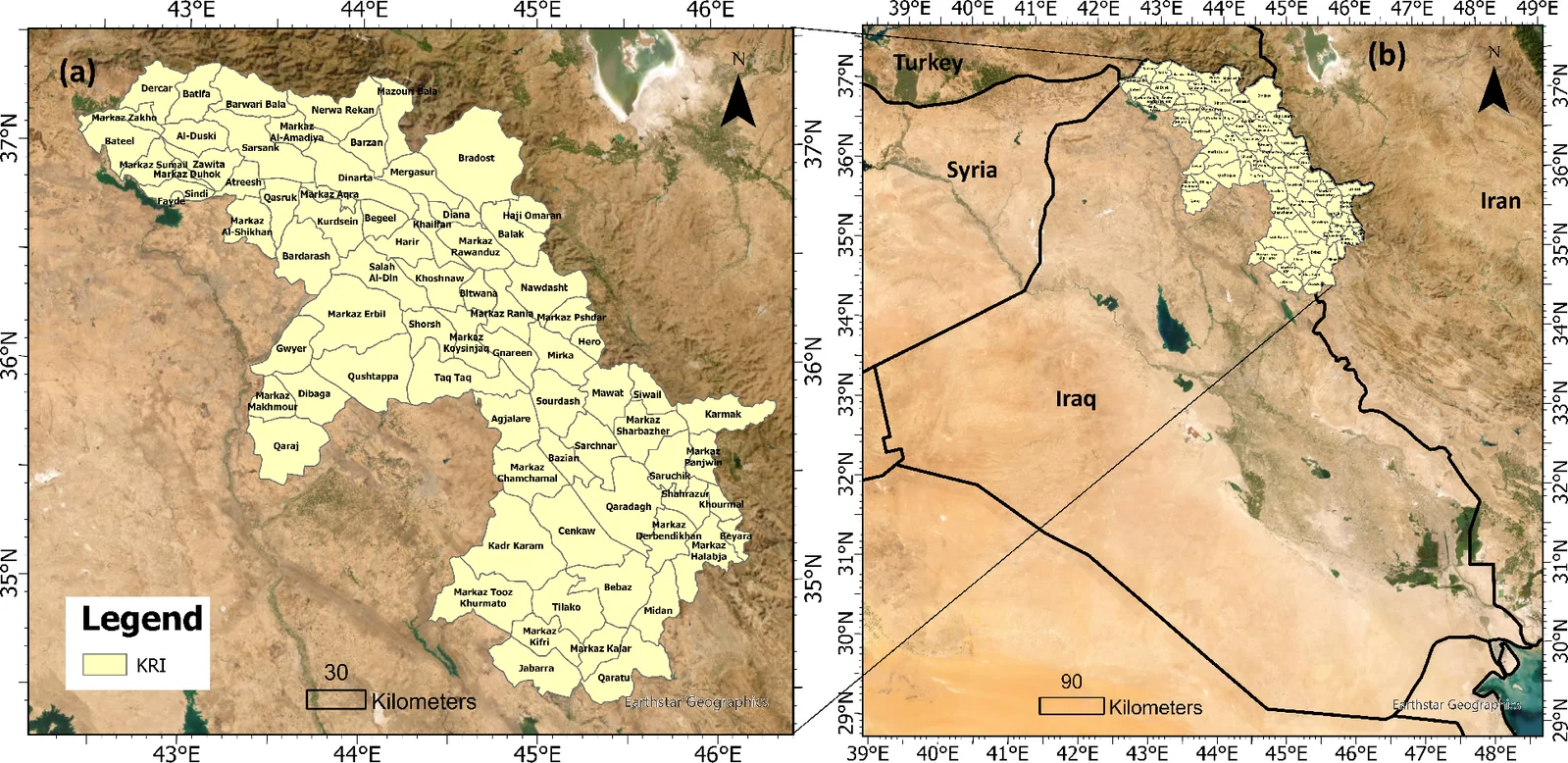
Location of the study area showing the Kurdistan Region in northern Iraq: (a) the KRI with its four governorates (Duhok, Erbil, Sulaymaniyah, and Halabja); internal thin lines represent district-level administrative boundaries; (b) regional setting of the KRI within Iraq and neighboring countries. Administrative boundaries were obtained from the Iraq Common Operational Datasets on the Humanitarian Data Exchange (OCHA; https://data.humdata.org/dataset/cod-ab-irq). The map was generated by the authors using ArcGIS Pro version 3.3 (Esri Inc., Redlands, CA, USA; https://www.esri.com/en-us/arcgis/products/arcgis-pro/overview). Basemap: Esri World Imagery. (sources: Esri, Maxar, Earthstar Geographics, and the GIS User Community).

The Kurdistan Region is located in northern Iraq and represents one of the country’s most seismically active regions due to its location within the active Arabia–Eurasia plate collision zone (Fig. 1). The region is characterized by complex geological structures, rugged topography, and a history of moderate to strong earthquakes, making it highly suitable for seismic hazard assessment. Administratively, the Kurdistan Region is divided into four governorates—Erbil, Sulaymaniyah, Duhok, and Halabja—which are shown in Fig. 1. These administrative units encompass the principal population centers and economic activities of the region and therefore represent areas where seismic hazards may have substantial societal and infrastructural impacts. Kurdistan region is vulnerable to seismic activity and notable earthquakes were recorded that have impact on local communities and infrastructures, because it’s located at the convergence of several tectonic plates^38^. The study area included the major cities with dense population and economic activities such as Sulaymaniyah and Erbil, which increase the potential impacts of seismic events^39^.

This study aims to develop a SHZ map for the KRI through the integration of geospatial datasets and GIS-based AHP techniques. Multiple geological and seismic conditioning factors were systematically analyzed to delineate the relative spatial variability of seismic hazard across the region. The contribution of this study is the methodological framework itself, multi-criteria AHP weighting combined with GIS-based weighted overlay and FR validation, is well established in the hazard zonation literature. The novelty of the present work is therefore regional and application-oriented rather than methodological: to the best of the authors’ knowledge, it provides the first regional-scale, screening-level seismic hazard zonation covering the entire Kurdistan Region of Iraq, a seismically active but under-studied segment of the Arabian–Eurasian convergence zone where no such region-wide zonation product previously existed. Additional contributions include the assembly of a harmonized, openly sourced multi-criteria geospatial database for the region and the transparent documentation of weights, ranks, consistency testing, sensitivity analysis, and validation, which together make the workflow reproducible and transferable to comparable data-scarce regions. The study is explicitly framed as a screening-level relative hazard zonation intended for regional planning and prioritization; it does not constitute a probabilistic or deterministic seismic hazard assessment and does not provide ground-motion parameters for engineering design. The findings deliver useful first-order information for structural engineers, urban planners, and policymakers to identify areas where detailed seismic investigations and mitigation planning should be prioritized.

## Methods and materials

### Study area

The geographical setting of the Kurdistan Region is presented in Fig. 1 (Introduction). The study covers the entire Kurdistan Region of Iraq, including the four governorates of Erbil, Sulaymaniyah, Duhok, and Halabja, which constitute the principal administrative units used for spatial analysis. The seismic hazard assessment was performed over the entire study area using the datasets and methodology described below.

### Geological and tectonic setting

The KRI occupies the northwestern (Iraqi) segment of the Zagros orogen, formed by the ongoing collision between the Arabian and Eurasian plates. GPS measurements indicate that the Arabian Plate moves northward relative to Eurasia at approximately 22 ± 2 mm/yr^40^, and in the northwestern Zagros this oblique convergence is partitioned into 3–6 mm/yr of belt-normal shortening accommodated by NW–SE-trending thrust and reverse faults, and 4–6 mm/yr of dextral (right-lateral) strike-slip motion on belt-parallel faults^41^, most notably the Main Recent Fault, for which a right-lateral slip rate of about 3 ± 2 mm/yr has been estimated^40^. The dominant structural grain of the region is therefore NW–SE, with fold axes and fault traces parallel to the orogen and tectonic transport directed toward the southwest.

**Fig. 2:**
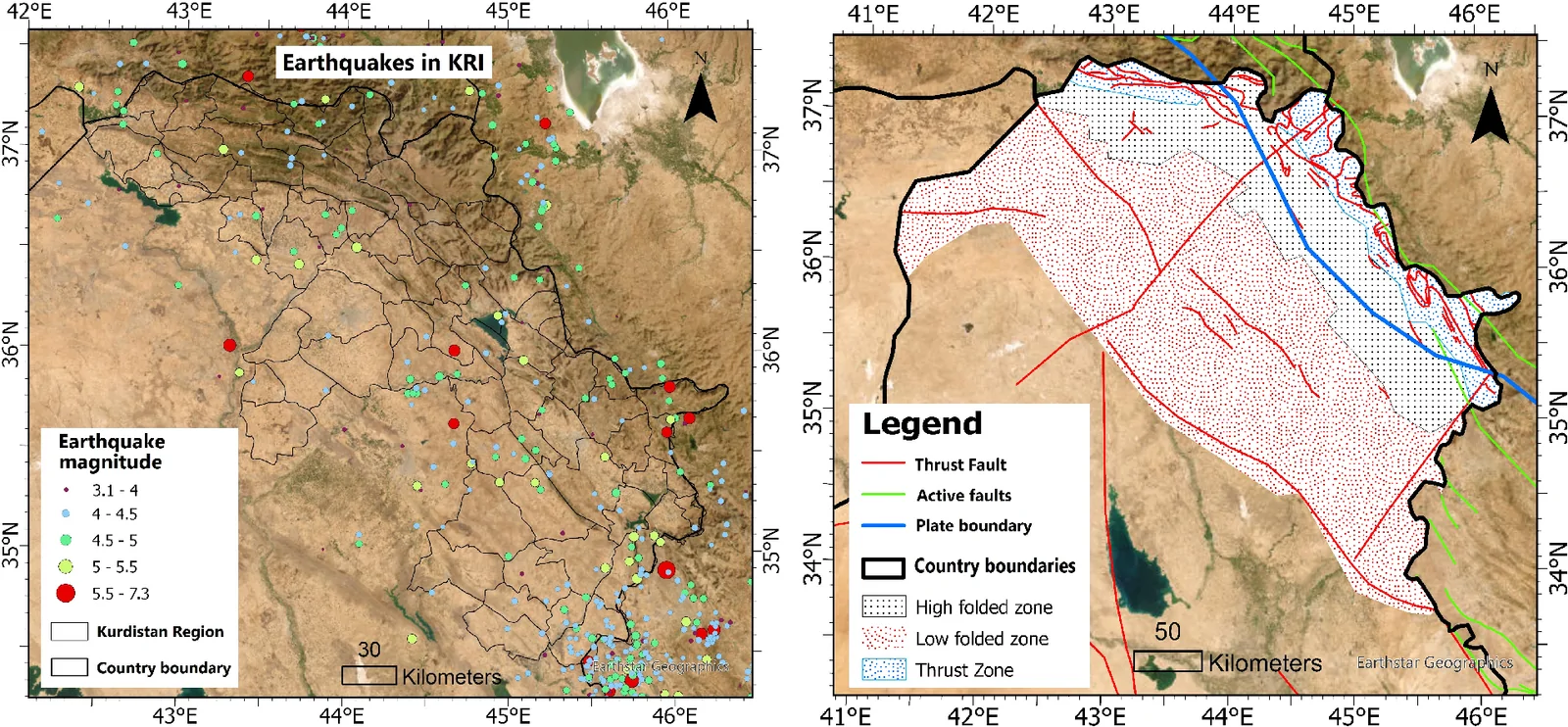
(a) Historical earthquakes (1917–2023) in the KRI and surrounding region; (b) regional tectonic framework showing thrust faults, active faults, the Arabian–Eurasian plate boundary, and the main tectonic zones of the Iraqi Zagros (Low Folded, High Folded, and Thrust zones). Tectonic zone boundaries were delineated by the authors from the geological dataset^45^, following the tectonic subdivision of Iraq^42^. The map was generated by the authors using ArcGIS Pro version 3.3 (Esri Inc., Redlands, CA, USA; https://www.esri.com/en-us/arcgis/products/arcgis-pro/overview). The basemap is from Esri World Imagery. (sources: Esri, Maxar, Earthstar Geographics, and the GIS User Community).

Following the tectonic subdivision of Iraq^42,43^, the KRI spans, from southwest to northeast, four principal zones of the Western Zagros Fold–Thrust Belt: (i) the Low Folded (Foothill) Zone, characterized by long-wavelength, low-amplitude anticlines developed in Cenozoic clastic sequences; (ii) the High Folded Zone, dominated by high-amplitude, short-wavelength, NW–SE- to E–W-trending anticlines exposing Mesozoic carbonate successions, bounded to the southwest by the Zagros Mountain Front Flexure; (iii) the Imbricate Zone, an intensely deformed belt of thrust sheets and tightly folded, faulted successions; and (iv) the Zagros Suture Zone along the Iraq–Iran border, comprising the Penjween–Walash, Shalair, and Qulqula–Khwakurk terranes, which contain ophiolitic, metamorphic, and radiolarite assemblages marking the closure of Neo-Tethys. The suture zone represents the surface expression of the Main Zagros Thrust, the fundamental, northeast-dipping boundary separating the Arabian Plate from the Iranian (Eurasian) terranes. Deformation intensity, structural complexity, and topographic relief all increase northeastward across these zones, and instrumental seismicity is correspondingly concentrated in the Imbricate and Suture zones and the adjacent High Folded Zone, the same northeast belt identified as high to very high hazard in the present zonation. The Imbricate and Suture zones are jointly represented as the Thrust Zone in Fig. 2.

Lithologically, the 26 formations mapped in the study area^44^ range from intensely deformed ophiolitic and metamorphosed units in the suture zone, through thick, competent Mesozoic platform carbonates in the High Folded Zone, to Cenozoic molasse-type clastics (e.g., Fatha, Injana, Mukdadiya, and Bai Hassan formations) and Quaternary alluvial deposits in the foothill plains and valleys of the central and southwestern KRI. This northeast-to-southwest transition, from deformed, faulted basement-proximal terranes to undeformed young sedimentary cover, constitutes the geological rationale underlying the lithology, fault proximity, and plate boundary proximity criteria used in the hazard model.

### Datasets

**Fig. 3:**
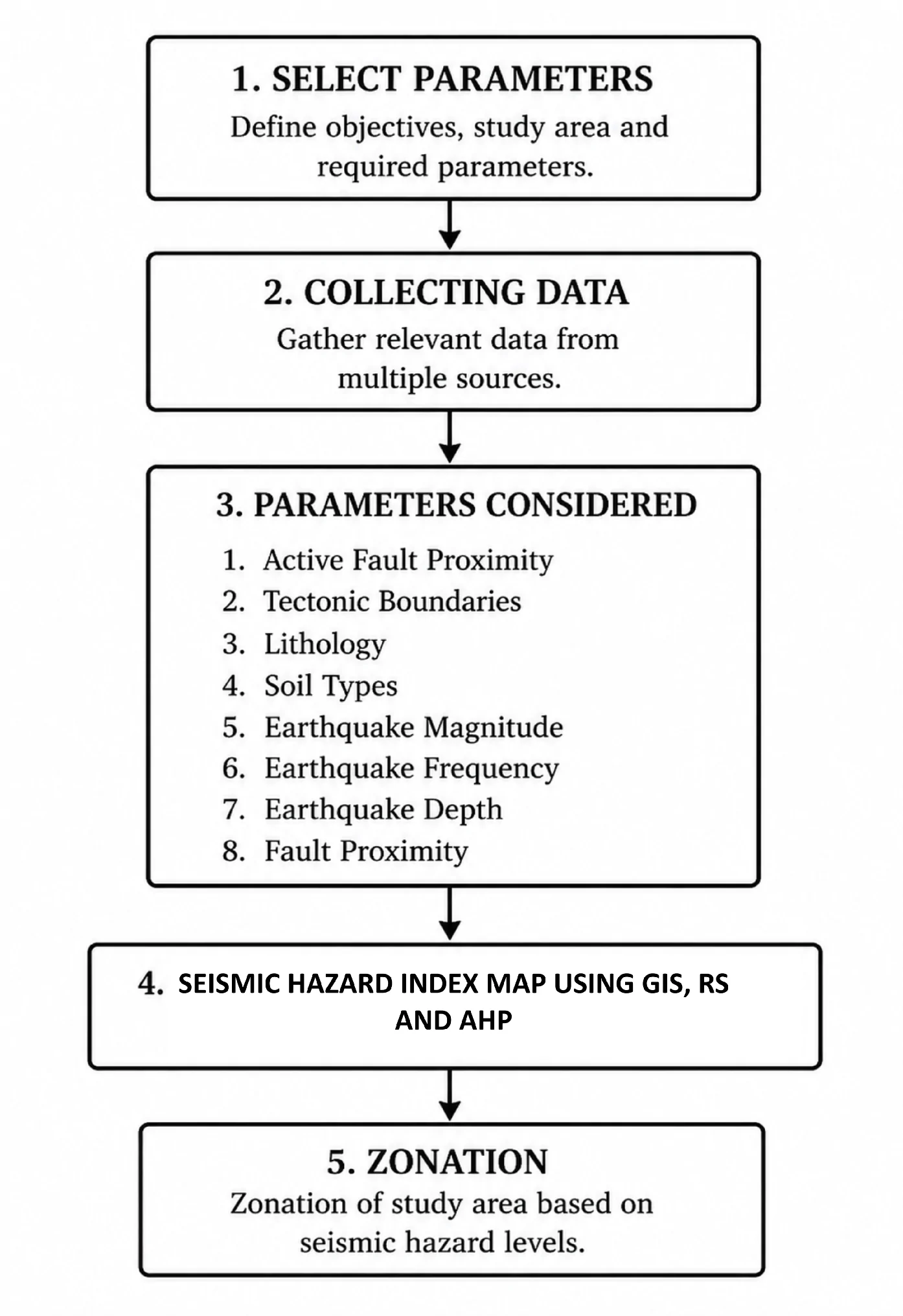
Methodological framework adopted for the seismic hazard zonation.

Based on the significance and data availability, 8 of the most earthquake hazard contributed parameters were selected in this study to investigate seismic hazard, such as active faults, thrust faults, plate boundary, lithology data, soil types, earthquake magnitude, earthquake frequency and depth. Historical earthquakes were collected for the study area from USGS (https://www.usgs.gov/) from 1917 to 2023 (Fig. 2a). The dataset of active faults from GEM global active faults^45^ and thrust faults^46^ were downloaded and prepared using ArcGIS Pro (Fig. 2b). Additionally, the digital model of plate boundaries was collected^47^. Moreover, the lithology data from FAO^48^ and soil map from Ministry of agriculture, Bagdad^49^ were collected for generating lithology and soil map for the study area. The detailed description of the used datasets was presented in Table 1. The flowchart of this investigation is presented in Fig. 3. The flowchart illustrates the sequential workflow followed in the study, including parameter selection, data collection, evaluation of seismic conditioning factors (active fault proximity, tectonic boundaries, lithology, soil types, earthquake magnitude, earthquake frequency, earthquake depth, and fault proximity), integration of Remote Sensing, GIS, and Analytic Hierarchy Process (AHP) techniques for seismic hazard vulnerability mapping, preparation of seismic hazard zonation maps for KRI, and subsequent accuracy assessment and result analysis.

**Table 1:** The characteristics of the dataset utilized in this research.

Parameters	Characteristics	Thematic map	Reference
Active fault gem	Shapefile	Active fault proximity	^45^
Thrust Faults	Shapefile	Thrust Faults proximity	^46^
Plate tectonic boundary	Shapefile	Plate tectonic proximity	^47^
Lithology types	Digital map	Geology	FAO^50^
Soil types	Digital map	Soil	Ministry of agriculture, Bagdad^51^
Earthquake data	Excel data (1917–2023)	Earthquake Magnitude density	https://www.usgs.gov/
		Earthquake Depth	
		Historical Earthquake density	

### Seismic hazard parameters

Based on the effective influence on seismic events and data availability, 8 parameters were considered to model the SHZ using MCDM techniques^23,52–54^. Factors such as (i) active fault proximity to active (ii) thrust Faults proximity, (iii) plate tectonic boundary proximity, (iv) lithology data, (v) soil types, (vi) earthquake magnitude, (vii) earthquake frequency, and (viii) earthquake depth were considered and thematic maps were generated in this study for KRI^55,56^.

### Analytical hierarchy process (AHP)

The Analytical Hierarchy Process (AHP) integrated with Remote Sensing (RS) and Geographic Information System (GIS) techniques was employed in this study to develop the Seismic Hazard Vulnerability Map (SHVM) for the Kurdistan Region of Iraq (KRI). The integration of these techniques provides a practical framework for screening-level relative seismic hazard zonation, particularly in regions characterized by complex tectonic settings and limited field-based geotechnical data availability. Remote sensing datasets provide reliable and spatially continuous information for seismic conditioning factors, whereas GIS enables efficient storage, spatial analysis, standardization, and overlay processing of multiple thematic layers. The AHP approach facilitates systematic weighting and prioritization of seismic hazard parameters according to their relative contribution to earthquake occurrence and hazard intensity AHP^57–59^.

The AHP method, originally proposed by Thomas L. Saaty, is one of the most widely used Multi-Criteria Decision-Making (MCDM) techniques for natural hazard zonation^57^. In the present study, the methodology consisted of four principal stages: (i) preparation of thematic layers, (ii) pairwise comparison of seismic hazard parameters, (iii) derivation of normalized weights and consistency evaluation, and (iv) weighted overlay integration in GIS to generate the SHVM.

Eight seismic conditioning parameters were considered in the AHP framework: active fault proximity, tectonic boundaries, lithology, soil types, earthquake magnitude, earthquake frequency, earthquake depth, and fault proximity. Each parameter was converted into raster thematic layers with a common spatial resolution and coordinate system in ArcGIS Pro. Subsequently, pairwise comparison matrices were constructed using Saaty’s nine-point scale, where values from 1 to 9 represent equal importance to extreme importance between two parameters.

The pairwise comparison matrix can be expressed as:

1 $$\:A={\left[{a}_{ij}\right]}_{n\times\:n}$$

where $\:{a}_{ij}$ represents the relative importance of parameter *i* compared to parameter *j*, and *n* is the total number of parameters. Reciprocal relationships were maintained such that:

2 $$\:{a}_{ij}=\frac{1}{{a}_{ij}}$$

The normalized comparison matrix was obtained by dividing each matrix element by the column sum:

3 $$\:{N}_{ij}=\frac{{a}_{ij}}{\sum\:_{i=1}^{n}{a}_{ij}}$$

The relative weight of each parameter was then computed using the arithmetic mean of normalized values:

4 $$\:{W}_{i}=\frac{\sum\:_{j=1}^{n}{N}_{ij}}{n}$$

where $\:{W}_{i}$ denotes the normalized weight of the *i*^th^ parameter.

To evaluate the reliability and logical consistency of expert judgments, the Consistency Index (*CI*) and Consistency Ratio (*CR*) were calculated. The maximum eigenvalue $\:\left({{\uplambda\:}}_{max}\right)$ of the comparison matrix was first determined, and the *CI* was computed as:

5 $$\:CI=\frac{{{\uplambda\:}}_{max}-n}{n-1}$$

The Consistency Ratio was then calculated using:

6 $$\:CR=\frac{CI}{RI}$$

where * RI* represents the Random Index corresponding to the matrix order *n*. According to Saaty’s criterion, the pairwise comparison matrix is considered acceptable when CR < 0.10. In the present investigation, the calculated *CR* value was 0.06, indicating satisfactory consistency and reliability of the assigned weights.

After determining the parameter weights, each thematic layer was reclassified into subclasses representing varying levels of seismic hazard contribution. Subclasses were assigned ranking scores according to their relative hazard significance. The weighted linear combination (*WLC*) approach was subsequently implemented in GIS to generate the Seismic Hazard Vulnerability Map using the following equation:

8 $$\:SHVM=\frac{\sum\:_{j=1}^{n}{W}_{i}{F}_{i}}{n}$$

where * SHVM* represents the Seismic Hazard Vulnerability Map, $\:{W}_{i}$ denotes the normalized weight of the *i*^th^ parameter, and $\:{F}_{i}$ represents the corresponding thematic factor layer.

The final SHVM was classified into different seismic hazard zones using GIS-based spatial analysis and zonal statistics tools in ArcGIS Pro. The classified hazard zones were subsequently utilized to prepare the Seismic Hazard Zonation (SHZ) map for the KRI. The methodological workflow ensured systematic integration of expert knowledge, geospatial datasets, and quantitative weighting procedures for regional-scale relative seismic hazard zonation.

### Accuracy assessment

To evaluate the reliability and predictive capability of the generated AHP–GIS-based Seismic Hazard Zonation (SHZ) map for the Kurdistan Region of Iraq (KRI), a quantitative accuracy assessment was conducted using the Frequency Ratio (FR) method and spatial proximity analysis of historical earthquake events. These approaches are widely used in natural hazard and seismic hazard assessment studies because they provide quantitative evaluation of the spatial relationship between observed hazard events and predicted hazard zones^60,61^. The FR method is particularly effective for assessing the correspondence between historical earthquake occurrences and classified hazard zones due to its simplicity, reliability, and statistical robustness^62^. Similar approaches have been successfully implemented in seismic hazard investigations and multi-hazard susceptibility studies worldwide.

For the FR analysis, an earthquake inventory database was prepared using historical earthquake events with magnitudes greater than or equal to 5.0 obtained from the United States Geological Survey (USGS). The earthquake inventory map was spatially overlaid with the generated SHZ map in the GIS environment. Subsequently, the spatial area corresponding to each hazard class (very low, low, moderate, high, and very high) was calculated, and the number of earthquake events falling within each class was determined.

The Frequency Ratio was calculated using the following equation^62^:

9 $$\:FR=\frac{\frac{E}{TE}}{\frac{P}{TP}}$$

where *E* represents the number of earthquake occurrences within a specific hazard class, *TE* denotes the total number of earthquake occurrences in the study area, *P* is the area of the corresponding hazard class, and *TP* represents the total area of the study region.

The FR method evaluates the spatial correlation between observed earthquake events and predicted hazard classes. An FR value greater than 1 indicates that earthquake occurrences are concentrated within a particular hazard zone more frequently than random spatial distribution, suggesting high susceptibility to seismic activity. Conversely, FR values lower than 1 indicate weaker spatial association and relatively lower seismic susceptibility. Therefore, a spatially consistent hazard zonation is expected to produce FR values greater than 1 for high and very high hazard classes, whereas low and very low hazard zones should exhibit FR values below 1.

In addition to FR validation, spatial proximity analysis was performed to further assess the effectiveness of the generated SHZ map. Historical earthquake records obtained from the USGS database were utilized for this purpose. Two earthquake datasets were considered: (i) major earthquake events with magnitude greater than 5.0, comprising 38 earthquake points, and (ii) an extended earthquake inventory containing magnitudes ranging from 3.4 to 7.3, comprising 333 earthquake events.

Buffer zones with a radius of 30 km were generated around the high and very high seismic hazard classes identified in the SHZ map. The number and percentage of earthquake events located within these buffered regions were subsequently calculated using GIS-based spatial analysis tools. The percentage of earthquake events within the buffer zones was determined as:

10 $$\:PE=\frac{{N}_{b}}{{N}_{t}}\times\:100$$

where *PE* represents the percentage of earthquake events within the buffered hazard zones, $\:{N}_{b}$ denotes the number of earthquake events located within the buffer region, and $\:{N}_{t}$ represents the total number of earthquake events considered in the analysis.

The proximity analysis was implemented based on the assumption that regions classified as high and very high seismic hazard zones should spatially correspond to areas with greater historical earthquake activity. Similar buffer-based spatial validation approaches have been widely applied in previous seismic hazard and natural hazard investigations to evaluate the spatial consistency between historical hazard events and predicted hazard zones^63^. Higher percentages of earthquake occurrences within the high-hazard buffer zones indicate stronger predictive performance and improved reliability of the AHP–GIS-based seismic hazard model. Comparable methodologies have also been successfully adopted in previous hazard susceptibility investigations to quantify the spatial agreement between observed hazard events and model outputs^64^.

### Sensitivity and uncertainty analysis

To evaluate the robustness and stability of the AHP-based seismic hazard zonation model, a sensitivity analysis was conducted by systematically perturbing the weights of individual seismic conditioning parameters. The analysis was performed to assess the influence of weight uncertainty on the final seismic hazard zonation results and to identify the most sensitive parameters controlling hazard classification. Each criterion weight was independently modified by ± 10% and ± 20% relative to its original normalized weight, while the remaining weights were proportionally adjusted to maintain the total weight sum equal to one. Following each perturbation scenario, the seismic hazard zonation map was regenerated using the weighted overlay approach in GIS.

The similarity between the original and perturbed hazard maps was quantitatively evaluated using multiple statistical indices, including Pearson correlation coefficient (r), mean absolute difference (MAD), percentage class change (PCC), and Jaccard similarity index (J).

The Pearson correlation coefficient was calculated as:

11 $$\:r=\frac{\sum\:_{i=1}^{n}\left({X}_{i}-\stackrel{-}{X}\right)\left({Y}_{i}-\stackrel{-}{Y}\right)}{\sqrt{\sum\:_{i=1}^{n}{\left({X}_{i}-\stackrel{-}{X}\right)}^{2}\sum\:_{i=1}^{n}{\left({Y}_{i}-\stackrel{-}{Y}\right)}^{2}}}$$

where $\:{X}_{i}$ and $\:{Y}_{i}$ represent the seismic hazard values of the original and perturbed maps, respectively.

The mean absolute difference was computed as:

12 $$\:MAD=\frac{1}{n}\sum\:_{i=1}^{n}\left|{X}_{i}-{Y}_{i}\right|$$

The percentage class change was determined using:

13 $$\:PCC=\frac{{N}_{c}}{{N}_{t}}\times\:100$$

where $\:{N}_{c}$ represents the number of cells with changed hazard classes and $\:{N}_{t}$ is the total number of cells.

The Jaccard similarity index was calculated to evaluate spatial agreement between high-hazard regions of the original and perturbed maps:

14 $$\:J=\frac{A\cap\:B}{A\cup\:B}$$

where *A* and *B* represent the high-hazard regions in the original and perturbed maps, respectively.

## Results

### Spatial distribution of seismic conditioning parameters

**Fig. 4:**
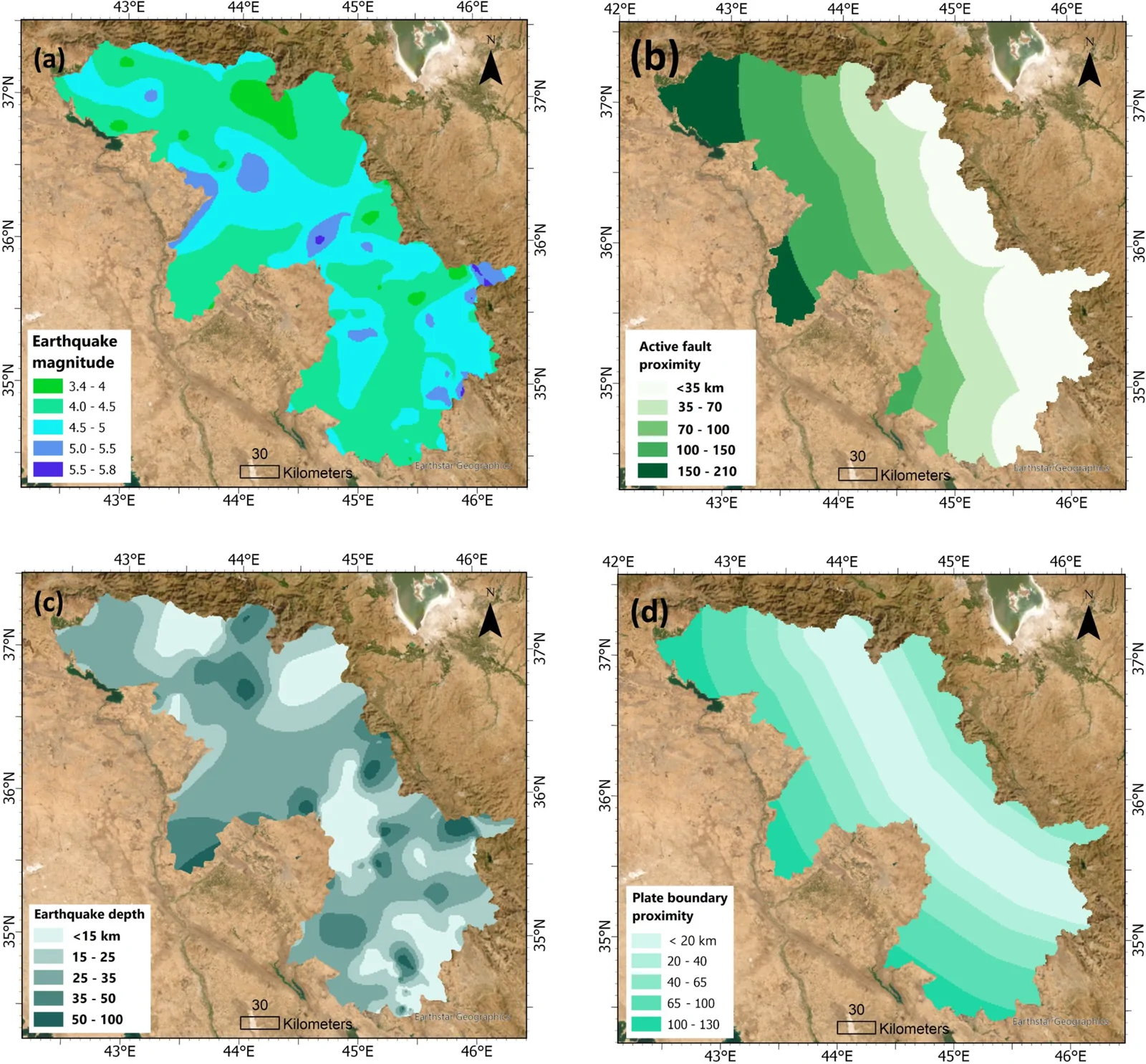

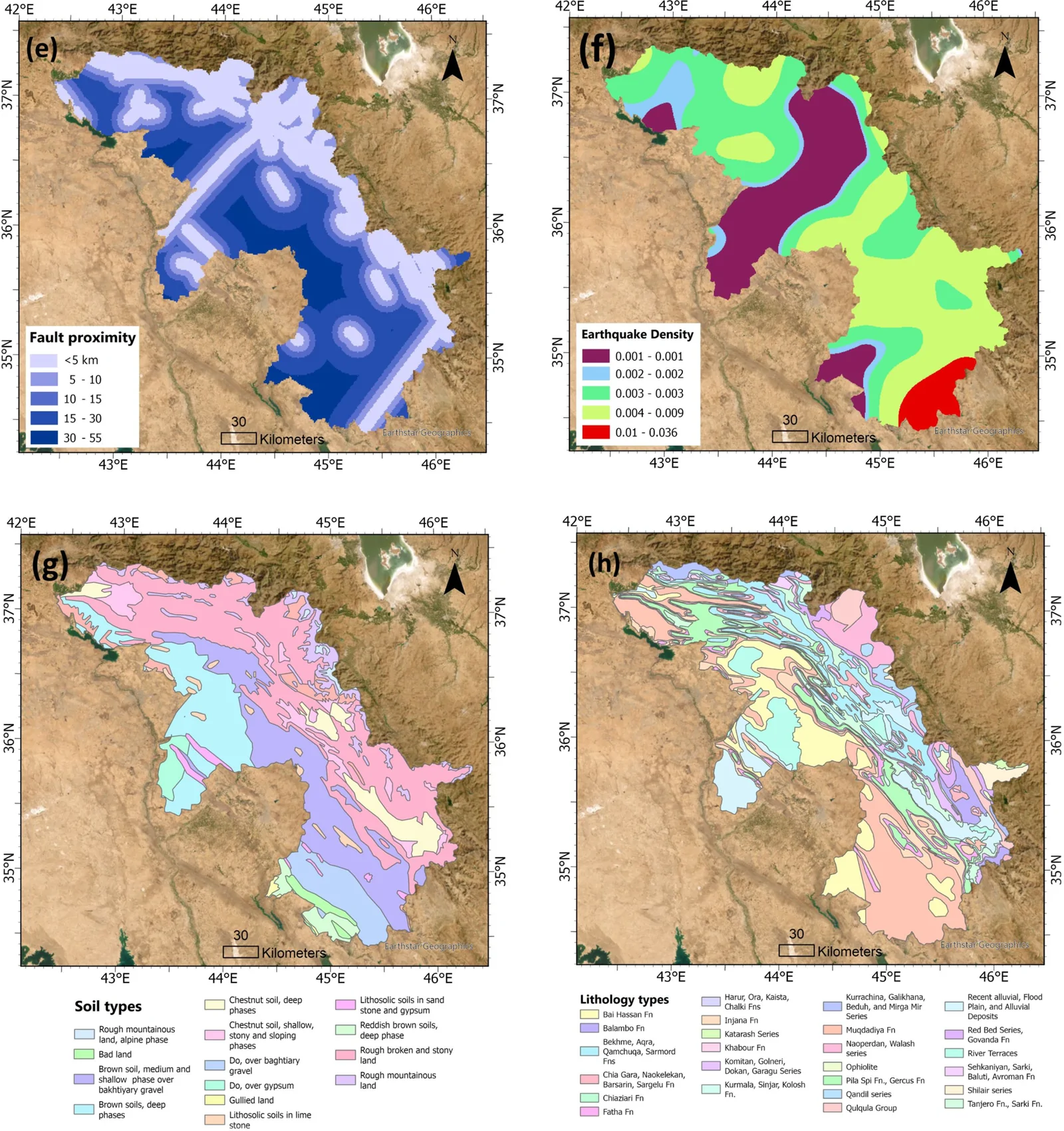
Seismic hazard zonation parameters: (a) Earthquake magnitude, (b) Active fault proximity, (c) Earthquake depth, (d) Plate boundary proximity, (e) Fault proximity, (f) Earthquake density, (g) Soil, and (h) Lithology. All maps were generated by the authors using ArcGIS Pro version 3.3 (Esri Inc., Redlands, CA, USA; https://www.esri.com/en-us/arcgis/products/arcgis-pro/overview), and the panels were compiled using Microsoft Paint (Microsoft Corporation; https://apps.microsoft.com/detail/9pcfs5b6t72h). Basemap: Esri World Imagery. (sources: Esri, Maxar, Earthstar Geographics, and the GIS User Community). Input datasets for each parameter are listed in Table 1.

The generated thematic layers revealed considerable spatial variability in seismic conditioning parameters across the Kurdistan Region of Iraq (KRI) (Fig. 4). The spatial distribution patterns demonstrate strong tectonic and geological controls on seismic hazard occurrence, particularly in the northeastern mountainous regions associated with the High Folded Zagros Zone. The active fault proximity and fault proximity maps indicate that the northeastern and eastern parts of the study area are located closer to major active tectonic structures and fault systems. These regions correspond to areas affected by the ongoing Arabian–Eurasian plate convergence and therefore exhibit relatively higher seismic susceptibility. In contrast, central and southwestern regions are situated farther from major active faults and consequently display comparatively lower tectonic influence.

The plate tectonic boundary proximity layer further highlights the tectonically active northeastern margin of the study area. Areas located near the tectonic collision zone exhibit shorter distances to plate boundaries and are therefore considered more vulnerable to seismic activity. The spatial distribution of tectonic boundaries closely corresponds with the distribution of historical earthquake events in the region. The earthquake magnitude thematic layer demonstrates that higher-magnitude earthquake events are predominantly concentrated in the northeastern mountainous belt of the KRI. Similarly, the earthquake frequency (earthquake density) map indicates higher concentrations of seismic events within tectonically active zones, while relatively lower earthquake densities occur toward the stable southwestern regions. These patterns confirm the strong relationship between regional tectonic activity and historical earthquake occurrence.

The earthquake depth distribution shows spatial variations in focal depth across the study area. Shallow to intermediate-depth earthquakes are mainly concentrated within the active tectonic zones of northeastern Iraq, whereas deeper events are relatively sparse and scattered. The concentration of shallow seismic events indicates elevated potential for stronger surface ground shaking in these regions. The lithological map revealed substantial geological heterogeneity within the study area, consisting of 26 geological formations with varying structural and lithological characteristics. Structurally complex and highly deformed formations are mainly distributed within the northeastern mountainous regions, whereas younger sedimentary and alluvial formations dominate the central and southwestern areas. Similarly, the soil-type map demonstrated spatial variability in soil characteristics across the region, with mountainous rocky soils dominating elevated terrains and alluvial or valley soils occurring in lowland regions.

Overall, the spatial distribution of seismic conditioning parameters indicates that the northeastern part of the Kurdistan Region exhibits higher seismic susceptibility due to the combined influence of active tectonic structures, high earthquake density, shallow seismic events, structurally complex lithology, and unstable mountainous terrain conditions.

### AHP weighting, pairwise comparison, and ranking results

The Analytical Hierarchy Process (AHP) was employed to determine the relative importance of the selected seismic conditioning parameters and to assign ranking values to each thematic subclass used in the seismic hazard zonation (SHZ) model for the Kurdistan Region of Iraq (KRI). The pairwise comparison matrix and the final normalized weights derived from expert judgment are presented in Table 2, while the ranking criteria for each parameter class are summarized in Table 3.

**Table 2:** The pairwise comparison matrix which was used to calculate the weights of considered parameters.

SHZ Parameters	Active Fault Proximity	Fault Proximity	Tectonic Plate Proximity	Earthquake Magnitude	Earthquake Depth	Lithology	Earthquake Density	Soil	Weight (%)
**Active Fault Proximity**	1	2	3	3	2	3	2	4	23
**Fault Proximity**		1	2	3	2	3	3	4	21
**Tectonic Plate Proximity**			1	2	2	2.5	3	3	16
**Earthquake Magnitude**				1	2	2	2	3	13
**Earthquake Depth**					1	1	1	1.5	7
**Lithology**						1	0.5	1	6
**Earthquake Density**							1	0.75	7
**Soil**								1	6

**Table 3:** The weights (W) of each seismic hazard factors and ranking (R) of each category.

SHZ Parameters	Class	*R*	W
Active fault proximity	< 35 km	5	23
	35–70	4	
	70–100	3	
	100–150	2	
	> 150	1	
Tectonic Plate Proximity	< 20 km	5	16
	20–40	4	
	40–65	3	
	65–100	2	
	100–130	1	
Earthquake Density	< 0.001	1	7
	0.001–0.003	2	
	0.003–0.004	3	
	0.004–0.01	4	
	0.01–0.036	5	
Soil	Rough broken and stony land	5	6
	Rough mountainous land, alpine phase	5	
	Gullied land	4	
	Bad land	4	
	Do, over gypsum	4	
	Do, over baghtiary gravel	4	
	Lithosolic soils in sand stone and gypsum	3	
	Lithosolic soils in lime stone	3	
	Chestnut soil, shallow, stony and sloping phases	3	
	Brown soil, medium and shallow phase over bakhtiyary gravel	2	
	Chestnut soil, deep phases	2	
	Brown soils, deep phases	2	
	Reddish brown soils, deep phase	1	
Fault proximity	< 5 km	5	21
	5–10	4	
	10–15	3	
	15–30	2	
	30–55	1	
Earthquake Depth	< 15 km	5	7
	15–25	4	
	25–35	3	
	35–50	2	
	50–100	1	
Lithology	Ophiolite	5	6
	Qulqula Group	5	
	Bekhme, Aqra, Qamchuqa, Sarmord Fns	5	
	Chia Gara, Naokelekan, Barsarin, Sargelu Fn	4	
	Harur, Ora, Kaista, Chalki Fns	4	
	Kurrachina, Galikhana, Beduh, and Mirga Mir Series	4	
	Kurmala, Sinjar, Kolosh Fn	3	
	Tanjero Fn., Sarki Fn	3	
	Balambo fn	3	
	Pila Spi Fn., Gercus Fn	3	
	Sehkaniyan, Sarki, Baluti, Avroman Fn	3	
	Chiaziari Fn	2	
	Fatha Fn	2	
	Katarash Series	2	
	Komitan, Golneri, Dokan, Garagu Series	2	
	Qandil series	2	
	Muqdadiya Fn	2	
	Bai hassan	2	
	Shilair series	2	
	Injana Fn	1	
	Khabour Fn	1	
	Recent alluvial, Flood Plain, and Alluvial Deposits	1	
	River Terraces	1	
	Red Bed Series, Govanda Fn	1	
	Naoperdan, Walash series	1	
	Naokelekan series	1	
Earthquake Magnitude	< 4	1	13
	4–4.5	2	
	4.5–5	3	
	5–5.5	4	
	5.5–5.8	5	

The consistency of the pairwise comparison matrix was evaluated using the Consistency Ratio (CR), which assesses the logical reliability of the expert-based weighting process. The calculated CR value was 0.06, which is below the acceptable threshold of 0.10 recommended by Saaty^65^, indicating satisfactory consistency and reliability of the assigned parameter weights.

The weighting results indicate that active fault proximity was the most influential parameter, receiving the highest normalized weight (23%). In the pairwise comparison matrix, active fault proximity was consistently ranked higher than most other parameters, including lithology, earthquake magnitude, and soil conditions, reflecting the dominant role of active tectonic structures in controlling seismic hazard occurrence. Regions located within 35 km of active faults were assigned the highest hazard rank (*R* = 5), whereas areas farther than 150 km were assigned the lowest rank (*R* = 1). This ranking structure reflects the increased probability of strong ground shaking and tectonic deformation near active fault systems.

Fault proximity received the second-highest weight (21%), further emphasizing the tectonic control on seismic hazard distribution within the KRI. Areas located within 5 km of mapped fault systems were assigned the highest susceptibility rank, while regions farther than 30–55 km were classified as low hazard zones. The pairwise comparison matrix demonstrates that fault proximity was considered more influential than earthquake density, lithology, earthquake depth, and soil conditions.

Plate tectonic boundary proximity obtained a weight of 16%, highlighting the importance of regional tectonic interactions associated with the Arabian–Eurasian convergent boundary. Regions located within 20 km of tectonic plate boundaries were assigned the highest rank (*R* = 5), whereas areas farther than 100–130 km received the lowest hazard ranking. The relatively high weighting of this parameter confirms the strong spatial relationship between seismicity and proximity to tectonically active plate margins.

It should be acknowledged that the three proximity-based tectonic criteria, active fault proximity, general fault proximity, and plate tectonic boundary proximity, are conceptually related and are expected to exhibit spatial correlation in the study area, since active faults in the KRI are largely concentrated along, and genetically linked to, the Zagros fold-and-thrust belt of the Arabian–Eurasian convergence zone. The three layers were nevertheless retained as separate criteria because they capture tectonic influence at different spatial scales and with different information content: plate boundary proximity represents the regional-scale convergence gradient, general fault proximity represents the density of all mapped structures including potentially reactivable inactive faults, and active fault proximity isolates structures with documented recent activity. However, their combined weight (60% of the total) means that the model is strongly, and partly redundantly, controlled by tectonic distance measures. This partial redundancy tends to reinforce, rather than distort, the northeast-to-southwest hazard gradient, but it also implies that the effective number of independent criteria in the model is smaller than eight, and that the hazard index is less sensitive to the non-tectonic parameters than the nominal weights suggest. This behavior is consistent with the sensitivity analysis, in which perturbations of the three fault- and boundary-related weights produced the largest, and spatially similar, changes in the hazard map.

Among the seismological parameters, earthquake magnitude received a moderate weight of 13%, indicating its substantial contribution to seismic hazard severity. Earthquake magnitudes between 5.5 and 5.8 were assigned the highest rank due to their greater destructive potential, while magnitudes below 4 were ranked as low hazard categories. Earthquake density and earthquake depth both received weights of 7%, indicating moderate influence on seismic hazard occurrence. Areas with high earthquake density values (0.01–0.036) and shallow earthquake depths (< 15 km) were assigned the highest hazard ranks because of their strong association with frequent and potentially damaging seismic events.

Lithology and soil conditions received comparatively lower weights (6% each), suggesting secondary but important contributions to local ground response and seismic amplification. Nevertheless, substantial variability was observed among lithological and soil subclasses. Highly fractured and tectonically deformed lithological formations, including ophiolite complexes, Qulqula Group, and Bekhme–Aqra–Qamchuqa formations, were assigned the highest hazard ranks (*R* = 5) due to their structural instability and association with active tectonic zones. In contrast, younger alluvial deposits, river terraces, and relatively stable sedimentary formations received lower hazard rankings.

Similarly, rough mountainous terrains, stony lands, gullied lands, and unstable lithosolic soils were ranked as highly susceptible because of their steep slopes, weak structural stability, and potential for seismic amplification and ground failure. Deep brown and reddish-brown soils located in relatively stable plains and valley regions were assigned lower hazard rankings.

The rank assignments for lithological and soil subclasses were guided by the well-established inverse relationship between material stiffness and seismic site amplification, whereby unconsolidated, weakly cemented, or intensely fractured materials exhibit lower shear-wave velocities and higher amplification potential than competent bedrock^66,67^. Accordingly, structurally deformed and fractured formations and weak, unconsolidated soil units were assigned higher hazard ranks, while consolidated, stable formations and deep, well-developed soils in low-relief terrain received lower ranks. It should be noted, however, that these ranks represent ordinal, expert-based proxies for relative ground response and are not calibrated against measured engineering parameters.

The pairwise comparison matrix, weighting framework, and ranking criteria collectively demonstrate that tectonic and fault-related parameters are the dominant controlling factors governing seismic hazard distribution in the Kurdistan Region of Iraq. Geological and soil-related parameters primarily contribute to modifying local seismic response and amplification effects. The derived parameter weights and ranking scores were subsequently integrated within the GIS environment using weighted overlay analysis to generate the Seismic Hazard Vulnerability Map (SHVM) and the final Seismic Hazard Zonation (SHZ) map for the study area.

### Seismic hazard zonation (SHZ)

**Fig. 5:**
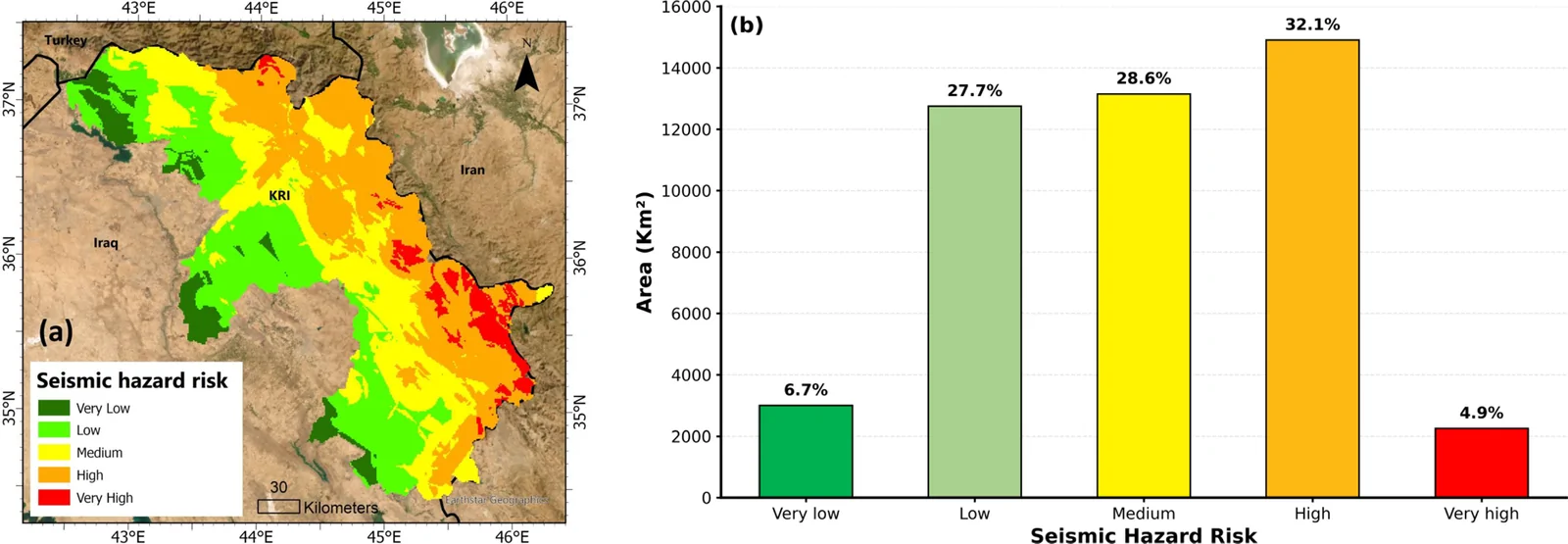
**(a)** Seismic hazard vulnerability map of the Kurdistan Region, Iraq; **(b)** Percentage and area (km²) of each seismic hazard class. The map in (a) was generated by the authors using ArcGIS Pro version 3.3 (Esri Inc., Redlands, CA, USA; https://www.esri.com/en-us/arcgis/products/arcgis-pro/overview). The chart in (b) was created by the authors using Microsoft Excel (Microsoft 365; Microsoft Corporation, Redmond, WA, USA; https://www.microsoft.com/en-us/microsoft-365/excel). Basemap: Esri World Imagery. (sources: Esri, Maxar, Earthstar Geographics, and the GIS User Community).

The generated Seismic Hazard Zonation (SHZ) map classified the Kurdistan Region of Iraq (KRI) into five seismic hazard categories: very low, low, moderate, high, and very high hazard zones (Fig. 5). The classification was performed using the weighted overlay results obtained from the integration of seismic conditioning parameters and their corresponding AHP-derived weights within the GIS environment. The categorization of the study area into distinct hazard classes provides a clearer understanding of the spatial variability of seismic vulnerability and earthquake susceptibility across the region.

The results indicate that the high seismic hazard class occupied the largest proportion of the study area. A total of 28 zones (36.4%) were classified as high seismic hazard regions, covering approximately 14,844 km^2^, which represents 32.1% of the total study area (Table 4). These regions are mainly concentrated in the northeastern mountainous parts of the KRI, corresponding to areas characterized by active tectonic structures, fault systems, and proximity to the Arabian–Eurasian convergent boundary. The dominance of the high hazard class indicates that a substantial portion of the region is exposed to elevated seismic risk conditions.

The moderate seismic hazard class represented the second-largest category, consisting of 19 zones (24.7%) and covering approximately 13,232 km^2^ (28.6%) of the study area. These regions generally occur in transitional tectonic and geological zones surrounding the highly active northeastern belt. Similarly, the low seismic hazard class also consisted of 19 zones (24.7%) and occupied approximately 12,798 km^2^, accounting for 27.7% of the total area. The low hazard zones are primarily distributed in relatively stable central and southwestern parts of the region where tectonic influence is comparatively weaker.

The very low seismic hazard class included 6 zones (7.8%) covering approximately 3,095 km^2^ (6.7%) of the study area. These regions are generally located farther from active faults and tectonic boundaries and are characterized by relatively stable geological conditions and lower historical earthquake activity. In contrast, the very high seismic hazard class occupied the smallest spatial extent, consisting of only 5 zones (6.5%) and covering approximately 2,284 km^2^ (4.9%) of the total study area. Despite its limited area, this class represents the most vulnerable and seismically active regions within the KRI. These areas are mainly concentrated near major active fault systems and tectonically deformed mountainous terrains where the combined effects of shallow earthquake activity, fault proximity, and structurally complex lithology significantly increase seismic susceptibility. The SHZ results demonstrate substantial spatial variability in seismic hazard distribution across the Kurdistan Region of Iraq. The predominance of moderate and high seismic hazard classes indicates that a considerable portion of the study area is potentially vulnerable to future seismic events.

**Table 4:** Distribution of seismic hazard zonation classes in the Kurdistan Region of Iraq.

Seismic hazard zonation	Number of zones in each class	%	Area (km^2^)	%
Very low	6	7.8	3095	6.7
Low	19	24.7	12,798	27.7
Medium	19	24.7	13,232	28.6
High	28	36.4	14,844	32.1
V high	5	6.5	2284	4.9

**Fig. 6:**
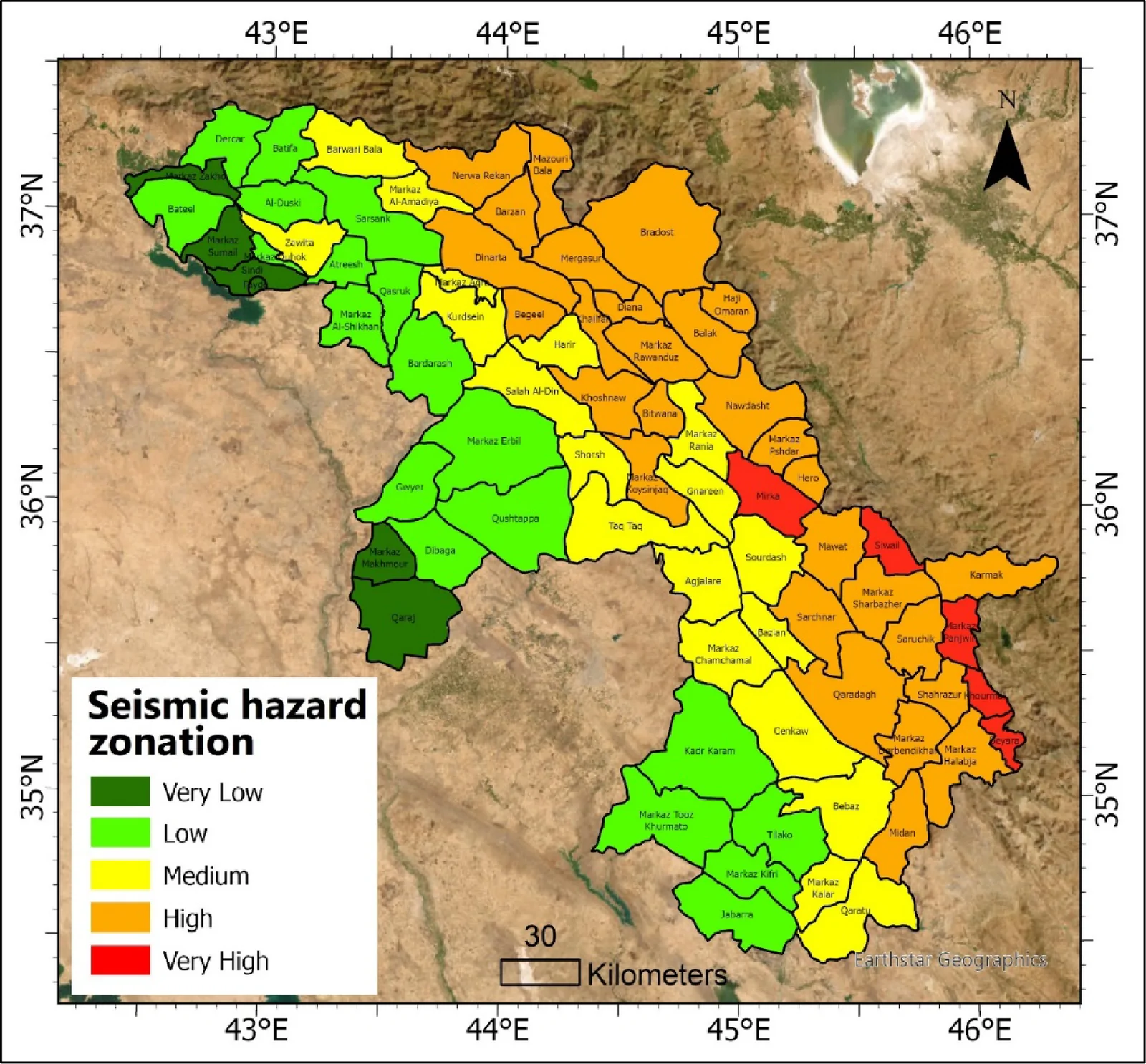
SHZ map of the different Kurdistan Region, Iraq. The map was generated by the authors using ArcGIS Pro version 3.3 (Esri Inc., Redlands, CA, USA; https://www.esri.com/en-us/arcgis/products/arcgis-pro/overview). Basemap: Esri World Imagery. (sources: Esri, Maxar, Earthstar Geographics, and the GIS User Community).

Figure 6. Seismic Hazard Zonation (SHZ) map of the different Kurdistan Region of Iraq (KRI) generated using the integrated AHP–GIS framework, illustrating the spatial distribution of five seismic hazard classes: very low, low, moderate, high, and very high hazard zones. The map highlights the dominance of moderate-to-high seismic hazard conditions in the northeastern and eastern mountainous regions associated with active fault systems and the Arabian–Eurasian tectonic convergence zone, while comparatively lower hazard levels are observed in the western and southwestern parts of the region.

These findings have critical implications for urban planners, engineers, and policy makers in KRI for better understanding the seismic events and robust frameworks of disaster management. And effective and proactive methodologies should be considered in regions which identified as high and very high zones for infrastructure resilience and early preparedness.

The zonation results translate into the following spatially explicit priorities. The very high hazard belt is concentrated along the Iraq–Iran border in Sulaymaniyah and Halabja governorates, encompassing the subdistricts of Penjwen, Beyara, Khourmal, Siwail, and Mirka. These border settlements should be treated as the first-order priority zone: here, seismic vulnerability assessments and retrofitting of schools, hospitals, mosques, and government buildings, enforcement of seismic provisions in new construction, and community-level earthquake preparedness programs are most urgently needed. The adjacent high hazard zone includes Halabja city, Darbandikhan, and the eastern districts of Sulaymaniyah Governorate, including Sulaymaniyah city — the region’s second-largest urban center. Critical infrastructure within this zone warrants particular attention; the Darbandikhan Dam, which sustained documented damage during the 2017 Sarpol-e Zahab earthquake, exemplifies the type of high-consequence facility for which detailed site-specific seismic safety re-evaluation should be prioritized. For the high hazard urban centers, priorities include updated building code enforcement, seismic microzonation, and contingency planning for the transport corridors linking Sulaymaniyah to Halabja, Penjwen, and the Bashmakh border crossing, which would be essential for post-earthquake emergency access. In the moderate hazard zones, including Erbil, the priority is progressive strengthening of code compliance for new high-rise construction, given the documented non-compliance of existing buildings in Erbil city^32^, combined with continuous seismic monitoring. In the low and very low hazard zones of the western KRI, including Duhok and Zakho, immediate structural intervention is less critical, and efforts can focus on baseline monitoring, awareness programs, and ensuring that regional emergency-response resources can be mobilized eastward toward the higher-hazard border districts when needed. This gradient-based allocation of mitigation effort, concentrating scarce technical and financial resources in the border subdistricts while maintaining proportionate measures westward, represents the principal practical application of the screening-level map, within the scope boundaries defined above.

GIS-based techniques aid significantly in enhancing the accuracy of seismic hazard analysis and playing critical role in giving insights for urban planners and policy makers for informed decision makings^68–70^. This study not only provide significant insights for better risk assessments, but also plays effective role in developing public safety campaigns and community preparedness initiatives and the resilience of the KRI against seismic threats^71^.

The results align with the historical records, as the one of the largest earthquakes was near the Halabja and Iraq-Iran border. In November 2017, and a devastating earthquake with 7.3 magnitude struck the Iran-Iraq border with its epicenter near Halabja region in KRI. The earthquake caused 630 deaths and 8100 injuries^72^. Additionally, multiple aftershocks were recorded after the first earthquake in 2018. These earthquakes occurred near the Iraq–Iran border, within the zones classified as high and very high hazard in this study. This spatial correspondence is encouraging; however, it should not be read as independent confirmation of model reliability, since the model is heavily weighted toward fault and plate-boundary proximity in precisely this area, and the 2017–2018 events form part of the catalog from which the seismicity-derived layers were constructed.

The soil and lithology data used in this study, were only included as a proxy indicator of relative site susceptibility, not as a measure of ground motion amplification. Based on investigations of related studies globally, the unconsolidated sediments, alluvial deposits, and soft soil layers are associated with increased seismic damage potential due to impedance contrast and site effects. This investigation utilized the regional geological and soil maps and reclassified the classes of each map into relative risk hazards based on its contribution to hazard risk depending on literature, which is a widely used methodology in AHP studies and susceptibility assessments in regions with limited data availability. Furthermore, the weights of the parameters do not represent the amplification factor, but shows the relative spatial susceptibility and the results highlight areas prone to higher seismic impact.

### Validation and accuracy assessment

**Fig. 7:**
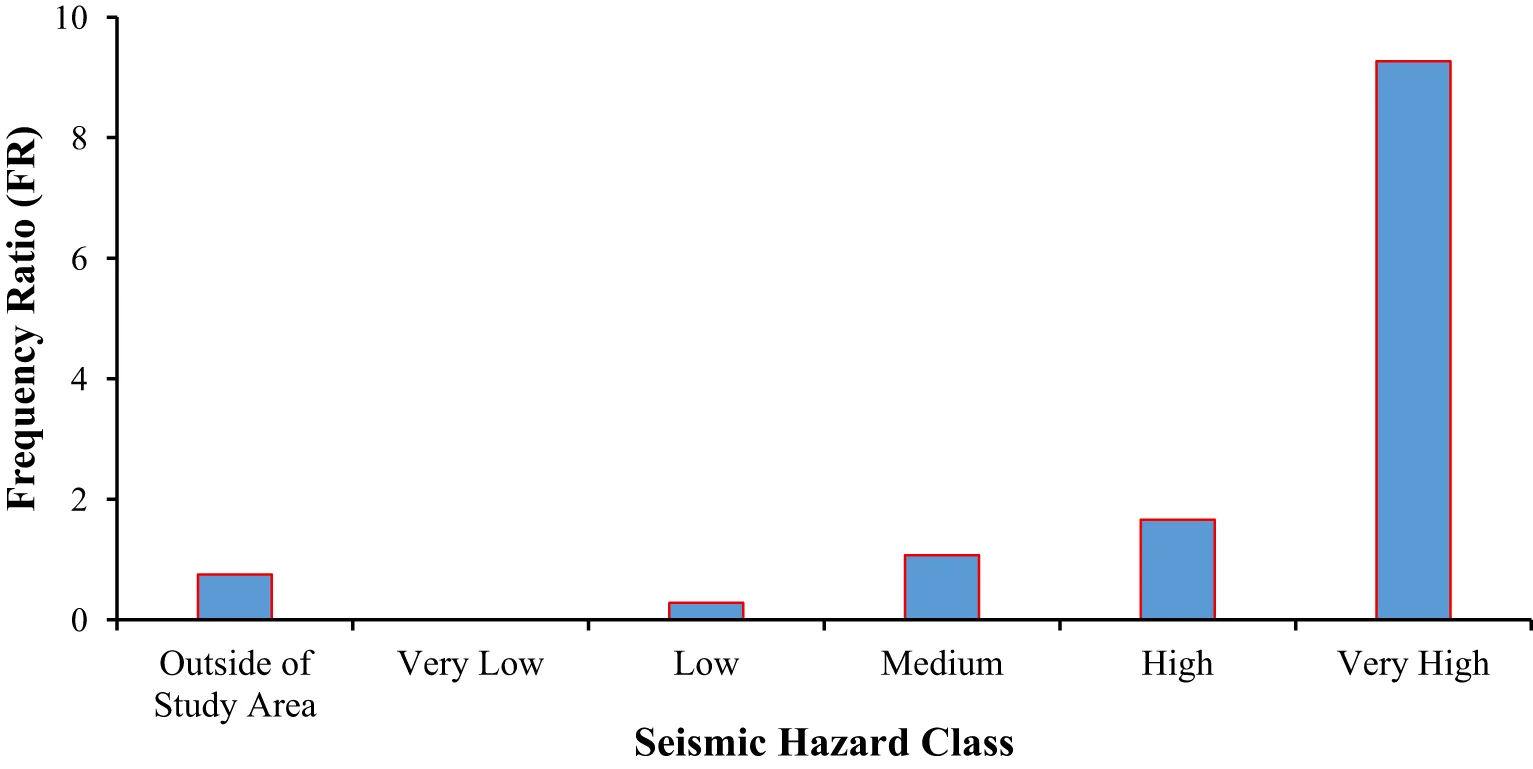
Frequency Ratio values for each SHZ.

The results of the accuracy assessment of FR techniques for each seismic hazard class: very low, low, moderate, high, and very high showed effective results. The FR outcomes for both very low and very high hazard classes were spatially consistent with areas of minimal and maximum recorded seismic activity in the study area, based on the spatial distribution of the historical earthquake events. The very low and low hazard class showed FR with less than 1, while the moderate hazard class had FR value around the 1, which shows the moderate level of earthquake events as expected (Table 5; Fig. 7).

The high and very high hazard classes yielded FR values greater than 1 (1.66 and 9.27, respectively), indicating a positive spatial association between these zones and the distribution of observed earthquake events. However, the exceptionally high FR value of 9.27 for the very high hazard class should be interpreted with caution. Because FR is computed as the ratio of the percentage of earthquake events to the percentage of class area, the very small areal extent of this class (1.96% of the study area) means that even a modest number of events produces a disproportionately large ratio; in this case, 6 of the 33 validation events (18.18%) fall within it. This value is further amplified by the strong spatial clustering of these events along the Iraq–Iran border zone, including the 2017–2018 Sarpol-e-Zahab sequence. Accordingly, the FR of 9.27 reflects a combination of genuine spatial correspondence, the small class area, and event clustering, and should not be overinterpreted as a direct measure of the predictive skill of the model, particularly given the partial dependence between the validation catalog and the model inputs noted above.

These results are consistent with previous studies that applied the FR technique to SHZ modeling and reported comparable spatial correspondence between AHP-GIS derived hazard classes and observed earthquake locations^73^. These results highlight the importance of considering multiple seismic-related criteria such as geology, soil, fault proximity and historical seismic activity in a multi criteria approach to model the seismic zonation hazard maps. The assessment of the results aids us in making informed decisions based on the results generated, specifically in classes that are identified as very highly susceptible to seismic activities.

**Table 5:** The results of FR for each seismic hazard class.

Hazard Class	No. of Total pixels	Total pixels (%)	Number of earthquakes (M > 5.0)	Number of earthquakes (%)	FR (Frequency Ratio)
Outside of study area	70,159	60.27%	15	45.45%	0.75
Very low	3,095	2.66%	0	0.00%	0.00
Low	12,798	10.99%	1	3.03%	0.28
Medium	13,232	11.36%	4	12.12%	1.07
High	14,844	12.75%	7	21.21%	1.66
Very High	2,284	1.96%	6	18.18%	9.27

**Fig. 8:**
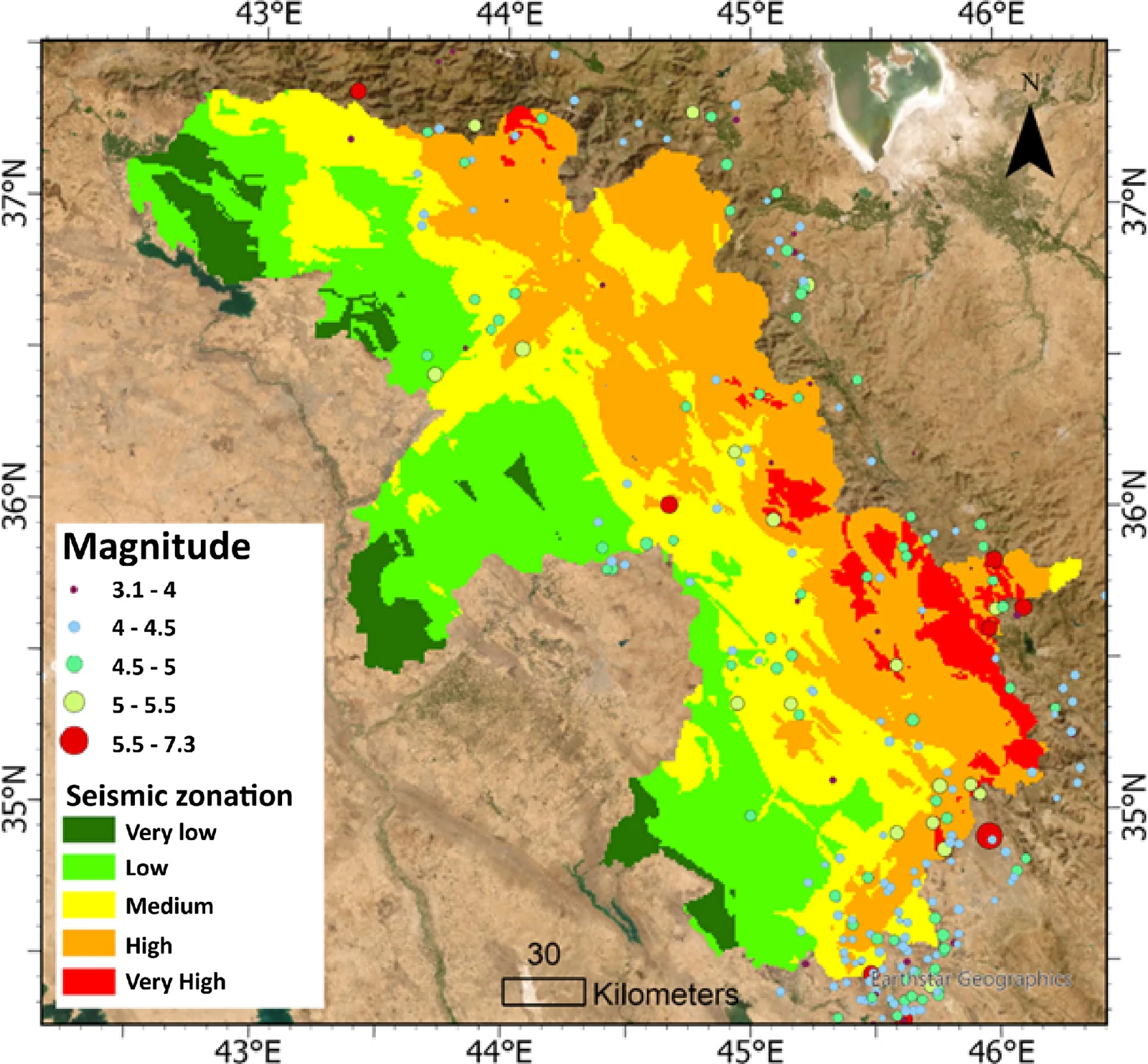
Spatial distribution of the earthquakes within 30 km around the study area. The map was generated by the authors using ArcGIS Pro version 3.3 (Esri Inc., Redlands, CA, USA; https://www.esri.com/en-us/arcgis/products/arcgis-pro/overview). Basemap: Esri World Imagery. (sources: Esri, Maxar, Earthstar Geographics, and the GIS User Community).

Moreover, the accuracy assessments based on the proximity analysis showed reasonable accurate outcomes for the AHP-GIS based model, specifically in high and very high hazard identified regions. For earthquakes with magnitude greater than 5, results showed that 78.9% of the earthquake events were located within 39 km of the high and very high identifies hazard classes. However, for all earthquakes from 3.4 to 7.3 magnitude, 77.2% of the events were located the 30 km radius of high and very high hazard zones, indicating strong spatial agreement between the modeled hazard classes and observed earthquake events (Fig. 8). The results of proximity analysis validate the performance of the generated model of this investigation considerably, which is comparable with similar studies in different parts of the world using the MCDM integration with GIS, supporting the validity of the utilized approach and weighting criteria^64,74^.

### Uncertainty analysis of the AHP-Based SHZ Model

To evaluate the robustness and stability of the developed AHP-based Seismic Hazard Zonation (SHZ) model, a sensitivity and uncertainty analysis was performed by perturbing the weights of individual seismic conditioning parameters by ± 10% and ± 20%. The analysis demonstrated that the generated SHZ model remained highly stable under all perturbation scenarios, indicating strong robustness of the adopted AHP weighting framework.

The Pearson correlation coefficients between the original and perturbed SHZ maps remained extremely high, ranging from 0.996 to 1.000 for all scenarios (Table 6). These results indicate a very strong spatial agreement between the original hazard zonation map and the modified models, even after systematic changes in parameter weights. Similarly, all perturbation scenarios produced statistically significant correlations (*p* < 0.001), confirming the reliability of the generated hazard maps.

**Table 6:** Sensitivity and uncertainty analysis of the AHP-based seismic hazard zonation (SHZ) model under ± 10% and ± 20% perturbations of conditioning factor weights.

Scenario	Criterion	perturbation_pct	original_weight	perturbed_weight	pearson_correlation	correlation_p_value	mean_abs_difference	pct_class_change	jaccard_high_hazard
active fault_−20%	active fault	−20	0.23	0.184	0.997	0	0.05	9.34	0.932
active fault_−10%	active fault	−10	0.23	0.207	0.999	0	0.025	4.21	0.971
active fault_+10%	active fault	10	0.23	0.253	0.999	0	0.025	5.54	0.957
active fault_+20%	active fault	20	0.23	0.276	0.998	0	0.05	10.17	0.93
fault proximity_−20%	fault proximity	−20	0.21	0.168	0.996	0	0.053	11.14	0.935
fault proximity_−10%	fault proximity	−10	0.21	0.189	0.999	0	0.027	5.87	0.97
fault proximity_+10%	fault proximity	10	0.21	0.231	0.999	0	0.027	5.93	0.954
fault proximity_+20%	fault proximity	20	0.21	0.252	0.996	0	0.053	10.16	0.929
plate techtonic proximity_−20%	plate techtonic proximity	−20	0.16	0.128	0.999	0	0.032	6.51	0.95
plate techtonic proximity_−10%	plate techtonic proximity	−10	0.16	0.144	1	0	0.016	3.19	0.973
plate techtonic proximity_+10%	plate techtonic proximity	10	0.16	0.176	1	0	0.016	4.2	0.963
plate techtonic proximity_+20%	plate techtonic proximity	20	0.16	0.192	0.999	0	0.032	7.26	0.941
magnitude_−20%	magnitude	−20	0.13	0.104	1	0	0.027	5.77	0.954
magnitude_−10%	magnitude	−10	0.13	0.117	1	0	0.013	3.31	0.97
magnitude_+10%	magnitude	10	0.13	0.143	1	0	0.013	2.95	0.978
magnitude_+20%	magnitude	20	0.13	0.156	1	0	0.027	5.54	0.963
earthquake depth_−20%	earthquake depth	−20	0.07	0.056	1	0	0.017	3.78	0.966
earthquake depth_−10%	earthquake depth	−10	0.07	0.063	1	0	0.009	2.36	0.974
earthquake depth_+10%	earthquake depth	10	0.07	0.077	1	0	0.009	1.6	0.992
earthquake depth_+20%	earthquake depth	20	0.07	0.084	1	0	0.017	3.07	0.976
lithology_−20%	lithology	−20	0.06	0.048	1	0	0.014	3.95	0.966
lithology_−10%	lithology	−10	0.06	0.054	1	0	0.007	2.83	0.977
lithology_+10%	lithology	10	0.06	0.066	1	0	0.007	0.81	0.994
lithology_+20%	lithology	20	0.06	0.072	1	0	0.014	2.11	0.984
earthquake density_−20%	earthquake density	−20	0.08	0.064	1	0	0.016	3.75	0.967
earthquake density_−10%	earthquake density	−10	0.08	0.072	1	0	0.008	2.22	0.977
earthquake density_+10%	earthquake density	10	0.08	0.088	1	0	0.008	2.09	0.992
earthquake density_+20%	earthquake density	20	0.08	0.096	1	0	0.016	3.64	0.979
soil_−20%	soil	−20	0.06	0.048	1	0	0.014	2.81	0.981
soil_−10%	soil	−10	0.06	0.054	1	0	0.007	1.52	0.993
soil_+10%	soil	10	0.06	0.066	1	0	0.007	2.25	0.975
soil_+20%	soil	20	0.06	0.072	1	0	0.014	4.11	0.967

Note: perturbation_pct = Percentage perturbation applied to the original parameter weight; original_weight = Original normalized weight assigned in the AHP model; perturbed_weight = Modified weight after perturbation analysis; pearson_correlation = Pearson correlation coefficient between original and perturbed SHZ maps; correlation_p_value = Statistical significance value (ppp-value) of the Pearson correlation; mean_abs_difference = Mean Absolute Difference (MAD) between original and perturbed hazard maps; pct_class_change = Percentage of pixels/cells with changed seismic hazard classes after perturbation; jaccard_high_hazard = Jaccard similarity index for high and very high seismic hazard zones between original and perturbed maps.

Among all parameters, active fault proximity and fault proximity exhibited the highest sensitivity to weight perturbations. Increasing the active fault weight by + 20% resulted in the highest percentage class change (10.17%), while decreasing fault proximity by − 20% produced the maximum class change of 11.14%. Likewise, perturbations in fault-related parameters generated comparatively larger mean absolute differences (MAD = 0.050–0.053) and lower Jaccard similarity values (0.929–0.935), indicating that fault-controlled parameters exert the strongest influence on seismic hazard classification within the study area (Table 6). These findings are consistent with the tectonic setting of the Kurdistan Region of Iraq, where active faults and structural deformation associated with the Arabian–Eurasian convergence predominantly control regional seismicity.

The tectonic boundary proximity parameter also demonstrated moderate sensitivity, with percentage class changes ranging from 3.19% to 7.26% and Jaccard similarity indices between 0.941 and 0.973 (Table 6). This further confirms the significant role of tectonic boundaries in controlling seismic hazard distribution across the region. In contrast, secondary conditioning parameters such as lithology, soil type, earthquake density, and earthquake depth showed comparatively lower sensitivity to weight perturbations. The perturbation of lithology and soil parameters generally resulted in low percentage class changes (< 4.11%) and high Jaccard similarity values (> 0.966), indicating that these factors have a relatively smaller influence on the overall SHZ pattern compared to primary tectonic controls (Table 6). Similarly, earthquake depth perturbations produced the smallest spatial variations, with percentage class changes as low as 1.60% and Jaccard similarity indices reaching 0.992, reflecting strong spatial consistency of the hazard model.

Earthquake magnitude and density exhibited intermediate sensitivity. Although these parameters contributed to local variations in hazard classification, the generated SHZ maps remained highly correlated with the original model (*r* = 1.000) and maintained high spatial similarity values (> 0.954) (Table 6). This indicates that the model is not excessively dependent on any single secondary seismic indicator.

The uncertainty analysis demonstrates that the developed AHP-based SHZ model is highly robust against moderate perturbations in parameter weights. The consistently high Pearson correlation coefficients, low mean absolute differences, and high Jaccard similarity indices indicate that the spatial distribution of seismic hazard zones remains stable under varying weighting scenarios. The results further confirm that fault-related parameters represent the dominant controlling factors for seismic hazard zonation in the Kurdistan Region of Iraq, while geological and environmental parameters provide complementary spatial refinement to the hazard assessment framework.

**Fig. 9:**
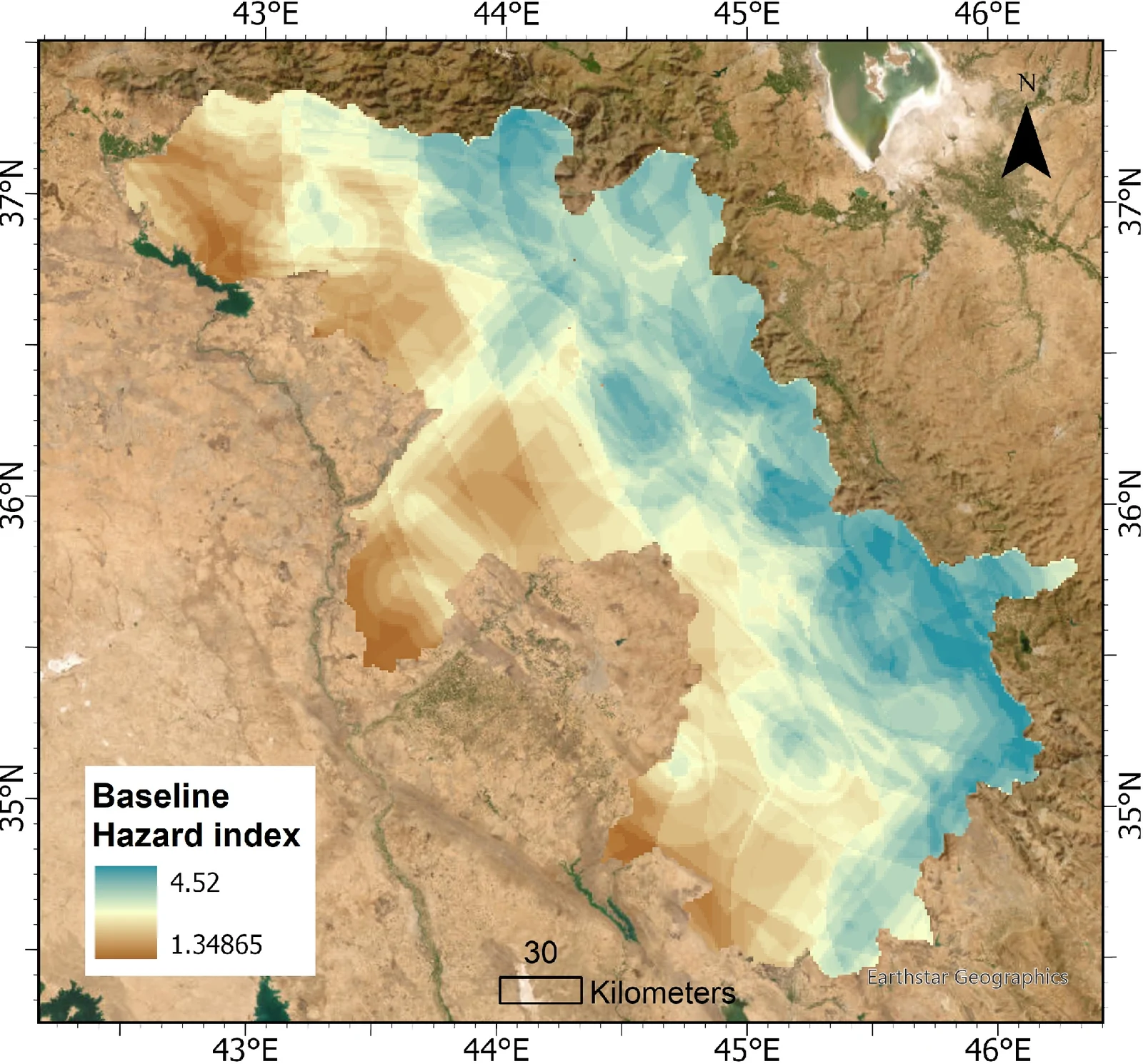
Baseline hazard index map used in sensitivity analysis. The map was generated by the authors using ArcGIS Pro version 3.3 (Esri Inc., Redlands, CA, USA; https://www.esri.com/en-us/arcgis/products/arcgis-pro/overview). Basemap: Esri World Imagery. (sources: Esri, Maxar, Earthstar Geographics, and the GIS User Community).

The baseline map using multiple quantitative indicators, including Pearson correlation of hazard index values, mean absolute difference, percentage of cells that changed hazard class, and Jaccard similarity of high and very high hazard zones (Fig. 9). The findings showed high level of stability across all sensitivity scenarios. Pearson correlation coefficients between the baseline and perturbed models ranged from 0.9959 to 0.9999, indicating strong agreement in hazard index values. Moreover, the mean absolute differences were consistently low, ranging from 0.007 to 0.053, emphasizing minimal numerical deviation from the baseline hazard index. Also, the spatial classification stability maintained, as the proportion of cells that changed hazard class varied between 0.81% and 11.14%, with an average of approximately 4.5%, suggesting that most areas retained their hazard classification under moderate weight perturbations. Notably, Jaccard similarity showed that the spatial extent of high and very high hazard zones remained highly consistent, as the values ranging from 0.93 to 0.99, with a mean value of 0.97.

It should be emphasized, however, that this sensitivity analysis evaluates only the internal stability of the model under moderate perturbations of the assigned weights, it does not eliminate the inherent dependence of the AHP framework on expert-based judgments. The perturbation scenarios test the robustness of the hazard patterns around the adopted weighting scheme, but the initial pairwise comparisons, and therefore the baseline weights themselves, remain subjective in nature.

The results indicated that the proposed GIS–AHP seismic hazard zonation is not overly sensitive to reasonable variations in criterion weights. The persistence of core high-hazard areas across all scenarios supports the robustness of the adopted weighting scheme and reinforces confidence in the spatial patterns identified by the baseline model.

**Fig. 10:**
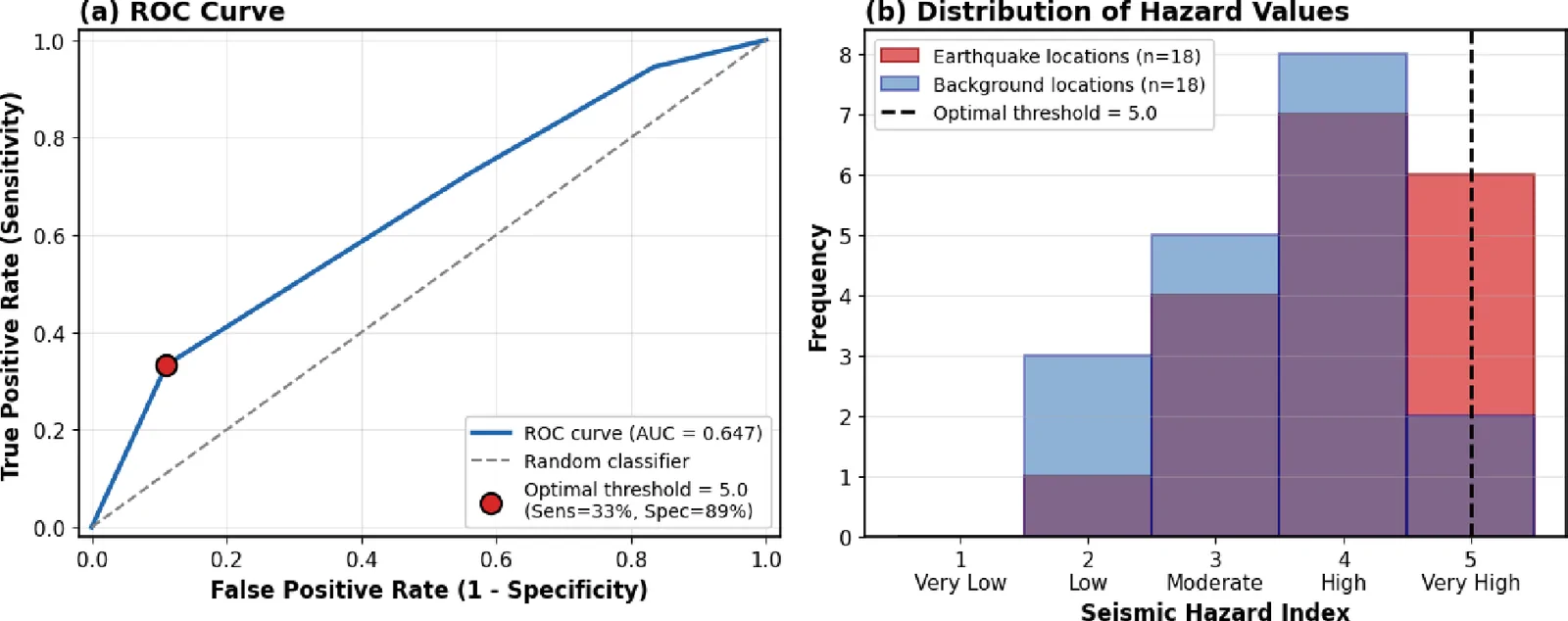
(a) ROC curve of the seismic hazard model showing an AUC of 0.647 and the optimal threshold (Hazard Index = 5.0), (b) Distribution of seismic hazard index values for earthquake and background locations, with the optimal threshold indicated by the dashed line. The charts were generated by the authors using Python (Matplotlib library) in Google Colaboratory (Google LLC; https://colab.research.google.com/).

To ensure accuracy and complete the frequency ratio and sensitivity analysis, the Receiver Operating Characteristic (ROC) curve analysis was performed to assess the spatial discrimination capability of the result of hazard map. Earthquake events with magnitude greater than 5.0 were selected to represent significant seismic occurrences. The occurred earthquakes inside the study area selected as the data for the analysis. moreover, equal number of points in the background were created within the study area to represent non-event locations. Finally, the Area Under the ROC Curve (AUC) was computed to calculate the ability of the hazard map to differentiate between earthquake and non-earthquake locations. The results showed satisfactory values with 64.7% (Fig. 10).

It is important to note that the validation results should be interpreted with caution due to a degree of circularity: three of the eight conditioning parameters (earthquake magnitude, earthquake density, and focal depth) were derived from the same USGS earthquake inventory (1917–2023) that was subsequently used as the reference dataset for the FR, proximity, and ROC-based validation. Because the validation events are not fully independent of the model inputs, the reported agreement partly reflects internal consistency of the framework rather than independent predictive performance. Accordingly, the moderate AUC value of 0.647 should be regarded as an indicative, screening-level measure of spatial correspondence rather than evidence of predictive skill.

## Discussion

The seismic hazard zonation results generated using the integrated AHP–GIS framework demonstrate strong agreement with the tectonic and geological characteristics of the Kurdistan Region of Iraq (KRI). The weighting and ranking structure adopted in the AHP model effectively captured the dominant controls governing seismic activity across the region. Among all conditioning parameters, active fault proximity and fault proximity received the highest weights because areas located near active tectonic structures are generally subjected to stronger seismic activity, higher ground shaking intensity, and greater crustal deformation. Similar findings were reported in previous regional and national seismic hazard investigations, where fault systems were identified as the primary controlling factors of earthquake occurrence and seismic vulnerability^75–77^. The dominance of fault-related parameters in the present study reflects the tectonic sensitivity of the KRI due to its location within the active Arabian–Eurasian convergent boundary system.

Plate tectonic boundary proximity also received a relatively high weight because earthquake events commonly occur along active plate margins and deformation zones. Northeastern Iraq lies within the High Folded Zagros Zone, which is characterized by active compression, folding, and faulting associated with the ongoing collision between the Arabian and Eurasian plates. Previous investigations similarly highlighted the significant role of tectonic boundaries in controlling seismic activity and regional earthquake distribution^78–81^. Therefore, the weighting assigned to tectonic boundary proximity is scientifically justified and consistent with regional tectonic conditions.

Among the seismological parameters, earthquake magnitude and earthquake depth were identified as important indicators of seismic hazard severity. Earthquake magnitude directly represents the amount of released seismic energy and is closely associated with earthquake intensity and structural damage potential. Similar GIS-based seismic hazard studies conducted in tectonically active regions also emphasized earthquake magnitude as a critical parameter in hazard zonation models^82,83^. Earthquake depth was assigned moderate importance because shallow-focus earthquakes generally generate stronger surface shaking and more severe infrastructure damage than deeper events. The incorporation of both earthquake magnitude and depth improved the representation of historical seismic behavior in the hazard assessment process.

Earthquake frequency was included to represent the spatial recurrence and density of seismic activity across the study area. Regions with repeated earthquake occurrences are generally considered more vulnerable to future seismic events. Previous studies, including Malakar and Rai^84^, similarly identified earthquake frequency as an effective indicator for identifying high-risk seismic zones. Although earthquake frequency received lower weight than fault-related parameters and magnitude, it still contributed significantly to the overall hazard evaluation.

Lithology and soil conditions were treated as secondary but important conditioning factors because geological formations and soil characteristics strongly influence seismic wave propagation, amplification, and ground response behavior. Different lithological units exhibit varying seismic responses depending on rock strength, weathering conditions, structural deformation, and fracturing intensity. Previous investigations demonstrated that hard, fractured, and mountainous lithological formations are generally associated with stronger seismic responses and higher instability^85,86^. In contrast, younger sedimentary deposits and relatively stable basin formations usually exhibit lower seismic susceptibility.

The qualitative ranking of lithological and soil subclasses was based on their expected contribution to seismic instability. Rough mountainous terrains, fractured bedrock, lithosols, and tectonically disturbed formations were assigned higher hazard ranks because steep slopes and structurally weak geological conditions can intensify seismic amplification and ground failure processes. Similar findings were reported by Dahal et al^87^. and Fatah et al^88^., who emphasized the vulnerability of fractured mountainous terrains to earthquake-induced instability. Conversely, stable valley soils and cohesive lowland formations received lower hazard rankings due to their comparatively lower contribution to seismic amplification and slope instability.

Lithological formations located near the High Folded Zagros Zone, including ophiolite complexes and the Qulqula Group, were ranked among the highest hazard classes because these formations are highly deformed and tectonically active under the ongoing Arabian–Eurasian collision regime. Geological investigations by Sissakian et al^89^. similarly identified northeastern Iraq as a structurally complex and highly fractured tectonic zone. Transitional formations such as Chia Gara, Sargelu, Harur, Ora, and Kaista were assigned intermediate hazard ranks because they exhibit moderate structural deformation and instability. In contrast, recent alluvial deposits were assigned lower hazard rankings due to their relatively stable basin environments and historically lower seismic activity levels^90^.

The generated SHZ map revealed that a substantial portion of the KRI falls within moderate-to-high seismic hazard classes, particularly in the northeastern and southeastern regions. These zones correspond closely with active tectonic structures, shallow earthquake activity, and highly deformed geological formations associated with the Zagros Fold–Thrust Belt. In contrast, relatively lower hazard zones were mainly distributed in central and southwestern areas where tectonic influence is comparatively weaker and geological formations are more stable.

The accuracy assessment results further demonstrated strong spatial agreement between the generated hazard zones and historical earthquake records. The Frequency Ratio (FR) analysis indicated that high and very high seismic hazard zones exhibited FR values greater than 1, confirming strong spatial correlation between historical earthquake occurrences and predicted hazard classes. Similarly, low and very low hazard zones generally produced FR values below 1, indicating weaker seismic association and validating the reliability of the hazard classification framework. The proximity analysis also demonstrated that most historical earthquake events were concentrated within or near the identified high-hazard regions, further supporting the predictive capability of the AHP–GIS model.

The sensitivity and uncertainty analysis confirmed the robustness of the developed SHZ model. The Pearson correlation coefficients between original and perturbed hazard maps remained extremely high (> 0.99) under all perturbation scenarios, while Jaccard similarity indices also indicated strong spatial consistency. Active fault proximity and fault proximity exhibited the highest sensitivity to weight perturbations, confirming that tectonic controls dominate seismic hazard distribution in the KRI. In contrast, lithology, soil, and earthquake depth showed relatively lower sensitivity, indicating that these parameters primarily influence local-scale hazard variability rather than regional seismicity patterns.

A comparison between the generated SHZ results and major historical earthquake events in the KRI provides a qualitative plausibility check of the zonation, although, as discussed below, this comparison does not constitute independent validation. One of the most destructive earthquakes affecting the region was the 2017 Sarpol-e Zahab earthquake, also known as the Halabja earthquake, which occurred on 12 November 2017 near the Iraq–Iran border with a magnitude of 7.3. The earthquake caused catastrophic damage across Iran and the KRI, resulting in approximately 630 fatalities and more than 8100 injuries overall^72,91^. Within the KRI, 9 fatalities and approximately 550 injuries were reported, along with severe damage to buildings and infrastructure, particularly near the earthquake epicenter^92^.

The southeastern parts of the KRI, especially Halabja and Darbandikhan towns, were among the most affected areas during the 2017 earthquake. These regions correspond closely with the high and very high hazard zones identified in the present AHP–GIS-based SHZ model. Darbandikhan, located approximately 40 km from the earthquake epicenter, was classified as a high hazard zone, and field investigations documented severe structural damage and building failures in the area. Similarly, Halabja city, located about 30 km from the epicenter, experienced strong ground shaking, infrastructure disruption, localized building damage, and electricity interruption^92^.

The spatial correspondence between the observed 2017 earthquake impacts and the high-hazard zones of the present model indicates that the zonation is consistent with the most damaging recent event in the region. Nevertheless, this agreement must be interpreted cautiously rather than as proof of predictive capability: the Halabja–Darbandikhan area lies close to the Zagros plate boundary and its associated active fault systems, which together account for 60% of the model weight, and the 2017–2018 Sarpol-e Zahab sequence is itself included in the earthquake catalog underlying the seismicity-derived layers. The agreement therefore partly reflects the model construction, in line with the structural circularity. What the comparison does demonstrate is internal coherence, the framework assigns its highest hazard levels to the area where the region’s most destructive documented earthquake actually occurred, which is a necessary, but not sufficient, condition for model adequacy.

A conceptual consideration inherent to the adopted framework should also be acknowledged. Because three conditioning layers (earthquake magnitude, earthquake density, and focal depth) are themselves derived from the historical seismicity record, the resulting hazard index is structurally predisposed to assign higher values to areas of past earthquake occurrence. Consequently, part of the observed agreement between the hazard zonation and the historical seismicity pattern is embedded in the model structure itself rather than emerging as an independent finding. This is a recognized characteristic of seismicity-informed multi-criteria zonation approaches, and it reinforces the interpretation of the resulting map as a relative, screening-level synthesis of tectonic and seismological evidence, in which the fault- and tectonic-related layers provide the forward-looking component, while the seismicity-derived layers encode the observed historical pattern.

Overall, the integration of expert knowledge, literature-based weighting, geospatial analysis, historical earthquake validation, and sensitivity assessment enabled the developed AHP–GIS model to coherently synthesize the seismic characteristics of the Kurdistan Region of Iraq. The generated SHZ map therefore provides a useful screening-level basis for prioritizing seismic risk mitigation efforts, land-use planning, and disaster preparedness within the region, pending confirmation through site-specific and probabilistic analyses.

To frame the scope of this work unambiguously: the final SHZ map is a screening-level, relative zonation product intended for regional-scale applications. It should be used for (i) first-order spatial prioritization of the KRI’s territory for seismic risk-reduction planning; (ii) identifying districts and corridors where detailed investigations, probabilistic seismic hazard analysis, site response studies, liquefaction assessment, and geotechnical characterization, should be undertaken first; (iii) supporting regional land-use planning, infrastructure corridor screening, and emergency-preparedness resource allocation; and (iv) raising institutional and public awareness of the pronounced northeast-to-southwest hazard gradient across the region. Conversely, the map should not be used for (i) estimating ground-motion parameters (e.g., peak ground acceleration or spectral accelerations) for structural design; (ii) site-specific engineering decisions, building code applications, or insurance rating; (iii) predicting the location, magnitude, or timing of future earthquakes; or (iv) parcel-level land-use decisions, given the regional resolution of the input datasets and the relative, ordinal nature of the hazard index. Within these boundaries, the map provides a transparent and reproducible basis for directing limited technical and financial resources toward the areas of greatest relative concern.

### Limitations and future research

Despite the effectiveness of the integrated AHP–GIS framework for seismic hazard zonation, several limitations should be considered. The AHP weighting process involves a degree of subjectivity because parameter weights are derived from expert judgment through pairwise comparisons. Although the obtained consistency ratio (CR = 0.06) indicates acceptable internal reliability, and the sensitivity analysis confirmed the stability of the hazard patterns under ± 10% and ± 20% weight perturbations, it must be acknowledged that this analysis only demonstrates the robustness of the model around the adopted expert-based weighting scheme, it does not remove the underlying subjectivity of the pairwise comparison process itself. Consequently, a differently constituted expert panel could produce a different baseline weighting, and the resulting zonation should be interpreted as conditional on the adopted expert judgments. Future work could reduce this dependence by employing data-driven or hybrid weighting approaches, such as frequency ratio-derived weights, machine learning-based feature importance, or fuzzy-AHP with multiple independent expert panels.

The developed seismic hazard zonation map represents a regional-scale relative hazard assessment and is not intended for site-specific engineering or microzonation applications. Detailed seismic design requires high-resolution geotechnical, geophysical, and strong-motion datasets, which were not available in the present study. In addition, the earthquake inventory obtained from the USGS database (1917–2023) may contain uncertainties related to incomplete historical records, particularly for older or lower-magnitude events, potentially affecting earthquake density and frequency representation.

In particular, the hazard ranks assigned to lithology and soil subclasses were not tied to measurable engineering parameters such as time-averaged shear-wave velocity in the upper 30 m (Vs30), site amplification factors, or geotechnical response properties, because in-situ measurements of these quantities are not available for the KRI at the regional scale. Their rankings therefore remain qualitative proxies for relative site response. Future refinements should incorporate proxy-based Vs30 estimates, such as topographic slope-derived Vs30^66^, geology-based site-condition mapping^67^, or field measurements including HVSR microtremor surveys and borehole geophysics, to replace the ordinal lithology and soil ranks with quantitative site-response parameters.

The adopted methodology provides relative seismic hazard assessment and does not incorporate probabilistic seismic hazard analysis (PSHA), ground motion prediction equations (GMPEs), peak ground acceleration (PGA), liquefaction analysis, or site response modeling. Therefore, the results should be interpreted as screening-level hazard zonation suitable for regional planning and prioritization rather than deterministic seismic prediction. Model validation was performed using frequency ratio and spatial proximity analyses, which assess spatial agreement with historical earthquake distributions but do not directly quantify predictive performance. Furthermore, the limited number of large-magnitude earthquake events (M > 5) may affect the stability of statistical validation metrics.

Moreover, the validation is partly circular, as the earthquake inventory used for validation is the same catalog from which the seismicity-related conditioning layers (magnitude, density, and depth) were derived; therefore, the validation metrics primarily confirm the internal spatial consistency of the model rather than its independent predictive capability. Future studies should address this by employing temporally split catalogs, spatial cross-validation, or independent seismic catalogs such as the ISC bulletin, to enable a genuinely independent evaluation of the model.

Furthermore, the multi-criteria framework did not include a formal multicollinearity assessment of the input layers. The three tectonic proximity criteria, in particular, are likely to be mutually correlated, introducing partial redundancy into the weighting scheme. Future applications should quantify inter-criteria correlation and, where strong redundancy is confirmed, either merge correlated criteria into a composite tectonic factor or adjust the AHP structure using a hierarchical (criteria–subcriteria) design that groups the proximity measures under a single tectonic criterion. Future studies should integrate site-specific geotechnical investigations, strong-motion recordings, probabilistic seismic analysis, and socio-economic vulnerability parameters to develop comprehensive seismic risk assessments for the Kurdistan Region of Iraq.

## Conclusions

This study demonstrated the applicability of the integrated GIS–AHP framework for regional-scale, screening-level relative seismic hazard zonation (SHZ) in the Kurdistan Region of Iraq (KRI). By incorporating tectonic, geological, and seismic conditioning parameters, the developed model delineated the relative spatial variability of seismic hazard across the region and highlighted the dominant influence of active faults, fault proximity, and tectonic boundary interactions associated with the Arabian–Eurasian convergence system. The generated SHZ map indicated that a substantial portion of the KRI falls within moderate-to-high seismic hazard classes, particularly in the northeastern and southeastern mountainous regions characterized by active tectonic deformation and shallow seismicity. The validation results demonstrated strong spatial agreement between the generated hazard zones and historical earthquake distributions, while acknowledging that part of this agreement is structurally embedded in the model, as seismicity-derived layers constitute three of the eight conditioning parameters, including the 2017 Sarpol-e Zahab (Halabja) earthquake, which severely affected Halabja and Darbandikhan areas identified by the model as high and very high hazard zones. The sensitivity and uncertainty analyses further confirmed the robustness of the developed AHP-based model, as the generated hazard patterns remained stable under different weighting perturbation scenarios.

Compared with probabilistic seismic hazard assessment (PSHA) approaches, the proposed GIS–AHP framework provides a computationally efficient and data-flexible alternative for regional hazard screening, particularly in data-scarce regions where detailed ground-motion records, geotechnical measurements, and probabilistic seismic datasets are limited. While PSHA models provide quantitative estimates of seismic exceedance probabilities and peak ground acceleration, the present approach effectively captures the relative spatial distribution of seismic susceptibility using integrated tectonic, geological, and historical seismic indicators. Therefore, the proposed framework can serve as an important preliminary tool for identifying priority areas requiring detailed probabilistic and site-specific investigations.

The generated hazard zonation map has important implications for urban planning, infrastructure management, and disaster risk reduction within the KRI. Areas classified as very high hazard zones, although limited in spatial extent, should be prioritized for detailed microzonation studies, geotechnical investigations, and stricter land-use regulations. Similarly, high hazard regions require reinforced seismic building codes, retrofitting of vulnerable structures, and targeted emergency preparedness strategies. Critical infrastructure, including hospitals, schools, dams, and transportation corridors, should receive particular attention in these regions. As emphasized in the Discussion, the map is intended solely for regional screening and prioritization, and not as a substitute for site-specific probabilistic or deterministic hazard analyses. The principal contribution of this work is thus regional and application-oriented, establishing the first region-wide screening-level SHZ baseline for the KRI, rather than methodological innovation. This study highlights the effectiveness of integrating geospatial techniques and multi-criteria decision-making methods for seismic hazard assessment in tectonically active regions. The developed framework provides a practical, transferable approach for regional screening-level hazard zonation and can be adapted to other earthquake-prone areas with limited seismic and geotechnical datasets.

## Data Availability

The datasets generated during and/or analyzed during the current study are available from the corresponding author upon reasonable request.
